# The transcriptome of circulating sexually committed *Plasmodium falciparum* ring stage parasites forecasts malaria transmission potential

**DOI:** 10.1038/s41467-020-19988-z

**Published:** 2020-12-02

**Authors:** Surendra K. Prajapati, Ruth Ayanful-Torgby, Zuleima Pava, Michelle C. Barbeau, Festus K. Acquah, Elizabeth Cudjoe, Courage Kakaney, Jones A. Amponsah, Evans Obboh, Anwar E. Ahmed, Benjamin K. Abuaku, James S. McCarthy, Linda E. Amoah, Kim C. Williamson

**Affiliations:** 1 Department of Microbiology and Immunology, Uniformed Services University of the Health Sciences, Bethesda, MD USA; 2 Noguchi Memorial Institute for Medical Research, University of Ghana, Accra, Ghana; 3 QIMR Berghofer Medical Research Institute, Brisbane, QLD Australia; 4 University of Cape Coast, Cape Coast, Ghana; 5 Department of Preventive Medicine and Biostatistics, Uniformed Services University of the Health Sciences, Bethesda, MD USA; 6 Present Address: University of Virginia, Charlottesville, VA USA

**Keywords:** Parasite biology, Parasite genomics, Biomarkers, Prognostic markers

## Abstract

Malaria is spread by the transmission of sexual stage parasites, called gametocytes. However, with *Plasmodium falciparum*, gametocytes can only be detected in peripheral blood when they are mature and transmissible to a mosquito, which complicates control efforts. Here, we identify the set of genes overexpressed in patient blood samples with high levels of gametocyte-committed ring stage parasites. Expression of all 18 genes is regulated by transcription factor AP2-G, which is required for gametocytogenesis. We select three genes, not expressed in mature gametocytes, to develop as biomarkers. All three biomarkers we validate in vitro using 6 different parasite lines and develop an algorithm that predicts gametocyte production in ex vivo samples and volunteer infection studies. The biomarkers are also sensitive enough to monitor gametocyte production in asymptomatic *P. falciparum* carriers allowing early detection and treatment of infectious reservoirs, as well as the in vivo analysis of factors that modulate sexual conversion.

## Abstract (web summary)

Malaria gametocytes are sexual-stage parasites transmitted from mammalian host’s blood back to their insect vector. Here, Prajapati et al. identify gametocyte-committed ring-stage biomarkers allowing to forecast malaria transmission potential.

## Introduction

Malaria transmission from human-to-human via a mosquito requires the production of sexual-stage parasites, called gametocytes. During each cycle of intraerythrocytic asexual replication, a subpopulation of parasites commits to sexual differentiation and produces gametocyte-committed (gc)-ring-stage parasites. In *Plasmodium falciparum*, the parasite responsible for the most virulent human malaria, the gc-ring matures over the course of ~8–12 days through five morphologically distinct stages (I–V) to become a mature, transmissible stage V gametocyte^1^. In vivo, immature gametocyte stages (I–IV) are sequestered primarily in the bone marrow^2–4^ and spleen^3^, and only terminally matured gametocytes (stage V) egress into the blood stream^5^. Therefore, circulation of stage V gametocytes in a patient’s peripheral blood only happens ~8–12 days after sexual commitment^6^ and currently serves as the only diagnostic stage for transmission potential. This delay in gametocyte detection after the initial commitment to sexual differentiation also makes it difficult to evaluate factors influencing the production of gc-rings in vivo as well as for early diagnosis and treatment of gametocytes before a person is infectious. In most locations, gc-rings are still effectively cleared by artemisinin derivatives. However, this activity against gc-rings is not universal among antimalarials acting against blood-stage parasites, such as antifolates and piperaquine^7^. In addition, drug sensitivity decreases rapidly as gametocytes mature and by stage V gametocytes are refractory to many antimalarials, with the notable exception of the 8-aminoquinolines: primaquine and the newly approved drug, tafenoquine. In Southeast Asia, reports of delayed parasite clearance following artemisinin-based combination therapy (ACT) administration have also been associated with increased gametocyte production^8^ consistent with increased survival of gc-rings, but without markers this could not be directly tested.

In vitro attempts to define early gametocyte markers by comparing the transcriptomes of culture-adapted gametocyte-producing and -deficient parasite lines identified genes expressed in early gametocytes (e.g., *pfs16, ge1, ge2, ge3, ge7, ge8, msrp1, pfgeco,* and *gexp5*)^9–15^. However, expression levels of most of these genes are low in early gc-rings and they continue to be expressed at some level in stage V gametocytes, which complicates their use as gametocyte-committed ring biomarkers^9^. Recently, an ex vivo gametocyte assay was developed that allows quantification of gc-rings in patient’s blood^16^. This approach identified asexual parasitemia, fever, parasite genotype, and possibly plasma lysophosphatidylcholine levels as factors that influence in vivo gametocytogenesis^16^.

To better understand the early stages of sexual differentiation in vivo and to develop molecular markers for gc-rings, this study examines the transcriptomes of uncomplicated malaria patients that had either high (H)-gametocyte-conversion rate (GCR) or low (L)-GCR in the ex vivo assay. Out of 18 genes overexpressed in gc-rings, 13 are consistently, significantly upregulated in H-GCR samples obtained from cross-sectional studies from two malaria seasons. *Ap2-g* (PFL1085w), *surfin 1*.*2* (PFA0650W), and *surfin 13.1* (PF13_0075) expression levels in gc-rings predict GCRs in samples from in vitro culture, uncomplicated malaria patients and human malaria volunteer infection study (VIS) subjects allowing their use as gc-ring biomarkers. We suggest gc-ring biomarkers are important tools to predict gametocyte production in submicroscopic to symptomatic infections ~8–12 days prior to circulation.

## Results

### Genes differentially expressed in vivo in gc-rings

Our recent demonstration of a wide range of gametocyte-conversion rates (GCR) in uncomplicated malaria patients^16^ provided the opportunity to identify genes differentially expressed in blood samples with H- and L-GCRs. In this prior study, an aliquot of blood was preserved for RNA isolation, and the rest was cultured ex vivo for 8 days in the presence of N-acetyl glucosamine to block asexual growth and allow GCR quantification. We selected a subset of these D0 blood samples from the 2016 cohort that had H-GCR (>5.5%) or L-GCR (0%) for transcriptome analysis (Supplementary Fig. 1a). Samples with H- (*n* = 8) or L- (*n* = 8) GCR also had high or low transcript levels of mature gametocyte-specific genes *pfs25* and *pfs230* in D8 ex vivo parasite RNA, respectively (Supplementary Fig. 1b). Mature gametocytes were not detected in any of the D0 samples by microscopy or RT-PCR analysis of *pfs25* transcripts (Supplementary Fig. 1c). All the selected samples had an initial parasitemia above 1.56% and other parameters such as range of D0 parasitemia, age, sex, hemoglobin levels, and WBC counts had a similar distribution in both groups (Supplementary Fig. 1c).

**Fig. 1:**
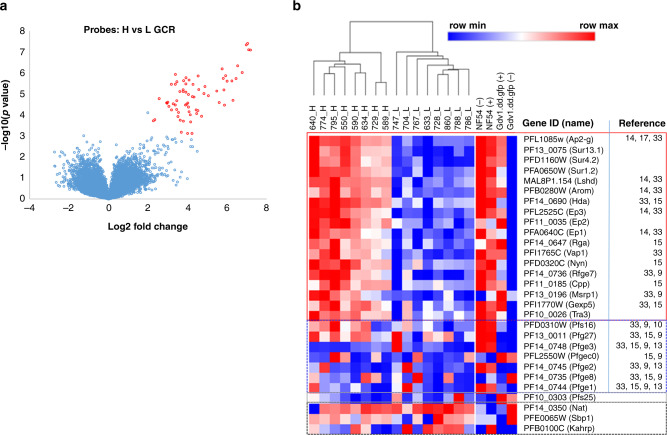
Transcriptome profiling of *P. falciparum* in vivo gametocyte-committed rings. **a** Volcano plot of the microarray signal difference between RNA from the high (H)- (*n* = 8) and low (L)- (*n* = 8) gametocyte-conversion rate (GCR) cohort D0 blood samples and the corresponding *P* value from a two-sided *t* test (*y* axis). Probes with > 2.0-fold differences (*x* axis) and a false discovery rate (FDR) ≤ 0.16 to control for multiple comparisons are shown in red. **b** Cluster analysis and heatmap of the relative expression of the indicated genes in both in vivo samples [H-GCR (sample#_H) and L-GCR (sample#_L) samples], and in vitro samples (gametocyte-producing lines, NF54 −/ + Shield 1 (Shld1) (−, + ) and *Pfgdv1.gfp.dd*+Shld1(+), and gametocyte-deficient line *Pfgdv1.gfp.dd -* Shld1 (-)). The relative expression of each gene is indicated by a color gradient from highest (dark red) to lowest (dark blue). The red box includes the 18 genes significantly upregulated in the H-GCR samples, the blue and gray boxes are previously reported early and late gametocyte-specific genes, respectively, and the black box contains ring-stage-specific genes. Reference to prior gene expression studies is indicated at the right^9,10,13–15,17,33^. Source data are provided as a Source Data file.

Transcriptome analysis revealed a significant increase in the transcript abundance of 18 genes (>2.0-fold) in D0 blood samples from the H-GCR (*n* = 8) compared to the L-GCR (*n* = 8) cohort (FDR < 0.16) (Fig. 1a). These genes were also more highly expressed in gametocyte-producing (wild-type, strain NF54 (wt) and *Pfgdv1.gfp.dd* parasites^16^ with stabilization ligand Shield 1 (Shld1) (~7–10% GCR)) than gametocyte-deficient (*Pfgdv1.gfp.dd* parasites without Shld1 (0.1% GCR)) lines (Fig. 1b). Cluster analysis using the expression profile of the 18 upregulated genes completely segregated the H-GCR samples (*n* = 8) from the L-GCR samples (*n* = 8) (Fig. 1b), indicating the consistency of the expression profile. In contrast, ring-stage-specific genes (e.g., *sbp1, nat*, and *kahrp*) show no differential expression between H- & L-GCR groups (FDR > 0.16). These transcriptomic data provide the first glimpse of the gene sets upregulated in circulating gc-rings, providing potential molecular markers as well as insight into the first hours of gametocyte differentiation. Of note, a number of early gametocyte genes identified in vitro were also not significantly upregulated in the H-GCR cohort (*n* = 8), suggesting that they may be expressed after sequestration. Current gene annotations divide the in vivo, gc-ring genes into five functional groups: (1) gene expression regulation, including the AP2 transcription factor required for gametocytogenesis (*ap2-g*)^17^, *hda, lshd, rga*, and *nyn*, (2) enzyme activity, *arom* (3) proteins predicted or known to be exported to the erythrocyte cytoplasm or surface, *ep1, ep2, ep3, surfin 13*.*1, surfin 1*.*2, surfin 4*.*2, ge7, gexp5*, and *vap1*, (4) putative invasion ligands, *tra*, and *msrp1*, and (5) conserved Plasmodium proteins (*cpp)*. To date, none of these genes have been reported to be specific for male or female gametocytes.

### In vivo gc-genes are regulated by AP2-G

**Fig. 2:**
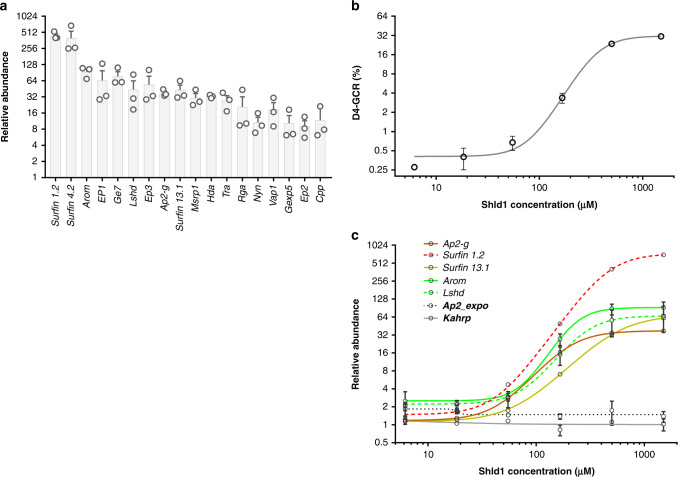
AP2-G augments expression of H-GCR-associated genes. **a** Transcript levels of the 18 genes upregulated in the high (H)- gametocyte-conversion rate (GCR) cohort were assayed in RNA from *E5.ap2-g.dd* parasites harvested (10–15 h) after one asexual cycle in the presence or absence of Shield 1 (Shld1). *Sbp1* RNA levels, which are high in both asexually and sexually committed ring stages, were used as the endogenous control, and *E5*.*ap2-g.dd* ring-stage parasites grown without Shld1 (−) were used as the reference line. The mean and standard error of the mean (SEM) for *n* = 3 biologically independent experiments are shown. **b**, **c** Shld1 dose-response curve of the day (D)4 GCR and gene expression. Ring-stage parasites were exposed to a range of Shld1 concentrations (0, 6.16 nM, 18.5 nM, 55 nM, 166 nM, 500 nM, and 1500 nM), and 10–15 h post invasion (hpi) after one asexual cycle an aliquot was isolated for RNA (**c**) and N-acetyl glucosamine was added to the remaining culture to block further asexual replication (**b**). The stage II–III gametocytemia on D4 was used to calculate the D4 GCR (**b**). **c** The fold change in gene expression between parasites treated with or without the indicated concentration of Shld1. *Kahrp* and *Ap2_expo* indicated in bold were included as asexual controls. The mean and range of *n* = 2 biologically independent experiments are shown for each Shld1 concentration in **b** and **c**. The profiles of the rest of the gametocyte-committed-ring genes are shown in Supplementary Fig. 2. Source data are provided as a Source Data file.

AP2-G is a critical regulator of gametocytogenesis and its presence is essential for the differentiation of gc-rings into early gametocytes^17^. Therefore, a gametocyte-inducible parasite line, *E5.ap2-g.dd*^17^ was produced by integrating a ligand-regulatable degradation domain (dd) in the frame at the 3′ end of the *ap2-g* coding region. In the absence of Shdl1 ligand, the presence of this dd domain targets AP2-G to the proteasome for degradation and blocks gametocytogenesis^18^. In contrast, the presence of Shld1 stabilizes AP2-G protein allowing gametocyte production. Using this system, we first evaluated the expression of all 18 genes in the presence and absence of Shld1 and demonstrate a Shld1-dependent increase in the transcript (Fig. 2a). To further evaluate whether the expression of all 18 genes correlated with gametocyte production, we regulated AP2-G protein levels by varying Shld1 concentration. As expected, there was a tight positive correlation between GCR and Shld1 concentration (*R*^2^ = 0.96) (Fig. 2b). Transcript levels for all 18 genes also showed a dose-dependent increase, which was in marked contrast to the RNA profile of the control genes, *kahrp* and *ap2-expo* (PF3D7_1466400), which are expressed regardless of sexual commitment (Fig. 2c and Supplementary Fig. 2). These results strongly support a gc-ring, AP2-G-dependent expression profile for these genes.

### Microarray validation using additional in vivo blood samples

**Fig. 3:**
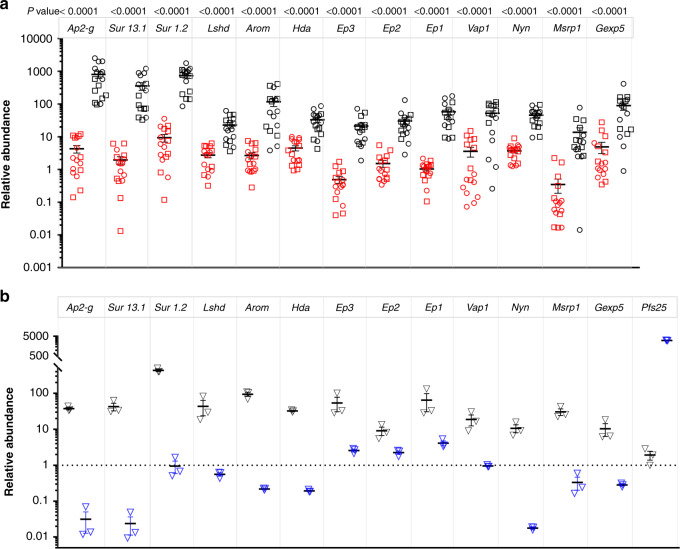
Validation of the differential expression of the genes in H- and L-GCR samples. **a** RNA levels of 13 genes were evaluated in an additional 32 day (D)0 blood samples from 2016 (circle) and 2017 (square) with high (H, black) and low (L, red) ex vivo D8 gametocyte-conversion rates (GCR). Differences between the biologically independent H- (*n* = 16) and L- (*n* = 16) GCR samples were analyzed using a two-sided Mann–Whitney test, and the *P* value indicated. The mean and standard error of the mean (SEM) are shown. **b** Transcript abundance in ring stage *E5.ap2-g.dd*+Shield 1 (Shld1) parasites (black triangle) and mature NF54 gametocytes (blue triangle). *Pfs25* transcript abundance was the control for mature gametocytes, and *sbp1* and *pf18s rRNA* were used as endogenous controls for ring stages and mature gametocytes, respectively. RNA from *E5.ap2-g.dd* (− Shld1) ring-stage parasites was used as the reference. The mean and SEM from *n* = 3 biologically independent samples are indicated. Source data are provided as a Source Data file.

We selected 13 genes for RT-qPCR validation of differential expression in the original D0 samples used for microarray analysis. All the genes were significantly upregulated in the H-GCR samples (Supplementary Fig. 3) and the expression-fold change ratio between the H- and L-GCR (H/L) samples ranged from 7.59 to 103, which confirms the microarray results. We then tested the expression levels of these genes in new sets of H- (*n* = 16) and L- (*n* = 16) GCR samples from the 2016 and 2017 cohorts (Supplementary Fig. 4a, b). Again in these D0 blood samples, all 13 genes were significantly overexpressed in the H-GCR group (Fig. 3a), and the expression-fold ratio of the genes in the H- and L-GCR (H/L) samples was >8.5, independently confirming the significant association of these genes with committed sexual rings in vivo. *Ap2-g* had the highest differential expression (187.8), followed by *surfin 1*.*2* (182.9) and *surfin 13*.*1* (79). We next evaluated in vitro the expression levels of these genes in stage V gametocytes that can also circulate in patient’s peripheral blood. The expression-fold ratios (ring/stage V) were highest for *surfin 13*.*1* (1785), *ap2-g* (1193), *nyn* (596), *surfin 1*.*2* (469), and *arom* (433), whereas the remaining genes ranged from 4 to 167 (Fig. 3b). Using these data coupled with consistently higher expression in D0 blood samples from the H-GCR cohorts in multiple years, we selected *ap2-g*, and *surfin 13*.*1* and *1.2* to test for the ability to predict GCR in vitro using several parasite strains with different in vitro GCR.

### Gc-ring biomarker development

**Fig. 4:**
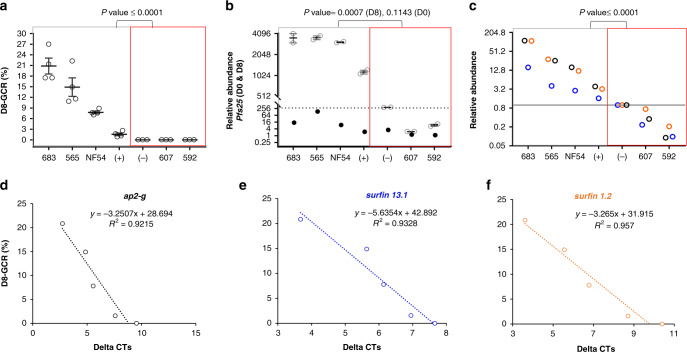
In vitro validation of the gc-ring biomarkers for gametocyte production. **a**–**c** Gametocyte-committed (gc)-ring biomarker expression was evaluated in six *P. falciparum* lines with a range of day (D)8 gametocyte-conversion rates (GCR). Each biologically independent parasite line (four clonal lines from recent field isolates (683, 565, 607, and 592), NF54, and the *Pfgdv1.gfp.dd* line cultured in the presence (+) and absence (−) of Shield 1 (Shld1)) was MACS/sorbitol synchronized. In the next cycle, 70% of the culture was collected for RNA analysis at 10–15 h post invasion (hpi) (D0) and N-acetyl glucosamine was added to the remaining culture. Gametocyte conversion was monitored on D8 by microscopy (**a**) and RNA (**b**). **a** The D8 GCR mean and standard error of the mean (SEM) of *n* = 4 independent experiments are plotted. **b** Transcript abundance of gametocyte-specific gene *pfs25* in D0 (filled circle) and in D8 (open circle) RNA. The D8 mean and range are indicated for two independent experiments with each independent parasite line, and the dotted line indicates the maximum transcript abundance of *pfs25* in the three low (L)-GCR lines. **c** The relative expression of *ap2-g* (black), *surfin 13*.*1* (blue), and *surfin 1*.*2* (orange) in D0 RNA in *n* = 7 biologically independent parasite lines. *Sbp1* transcript levels were the endogenous control, and RNA levels in *Pfgdv1.gfp.dd* (−) were the reference. Differences between the high (H, *n* = 4)- and the L-GCR (*n* = 3) biologically independent parasite lines were analyzed using a two-sided Mann–Whitney test (**a**–**c**), and the *P* values indicated. **d**–**f** D8 GCR correlation with the ∆Ct of *ap2-g* (**d**), *surfin 13.1* (**e**), and *surfin 1*.*2* (**f**). ∆Ct was calculated using *sbp1* as the control gene. The linear trend line for *n* = 5 independent parasite lines, all four H-GCR lines (683, 565, NF54, and Pfgdv1.gfp.dd+Shld) and the reference L-GCR line, (Pfgdv1.gfp.dd -Shld1), is indicated with its equation and the *R*^2^ value. Source data are provided as a Source Data file.

Expression levels of gc-ring biomarkers were analyzed in seven parasite lines with distinct gametocyte production profiles, wt NF54, *Pfgdv1.gfp.dd* in the presence and absence of Shld1, and four lines cloned from recent field isolates with high (sample id: 683 and 565) and low (592 and 607) gametocyte-conversion phenotypes as depicted in Supplementary Fig. 5. Gametocyte production varied in the cultures when analyzed by both the microscopic GCR (Fig. 4a) and D8 *pfs25* RNA levels, as did D0 RNA levels of *ap2-g, surfin 13.1,* and *surfin 1*.*2* (Fig. 4b–c). RNA levels of all three genes on D0 strongly correlated with the GCR and D8 *pfs25* RNA level (Supplementary Fig. 6a, b), and all three genes were also tightly co-expressed (*R*^2^ = 0.99) (Supplementary Fig. 6c, d). We further sought to determine if the difference in cycle threshold (ΔCt) between the Cts for the biomarkers and *sbp1* could be used to predict gametocyte production. This approach would allow the analysis of field samples without using a reference sample that might not be readily available. The ΔCt of each biomarker showed a tight correlation with D8 GCR (Fig. 4d–f) and the trend line of the graph provided an equation to predict GCR.

### Validation of the gc-ring biomarker prognostic efficacy

**Fig. 5:**
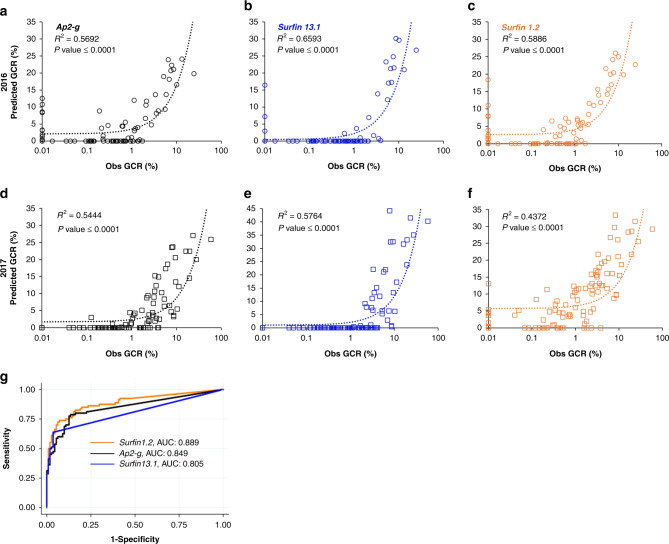
Correlation between predicted GCR and ex vivo observed GCR. The observed (Obs) gametocyte-conversion rate (GCR) was determined microscopically in the day (D)8 ex vivo cultures and the predicted gametocyte-conversion rate (GCR) was calculated for each gametocyte-committed (gc)-ring biomarker (*ap2-g* (black), *surfin 13.1* (blue), and *surfin 1.2* (orange)) in biologically independent D0 patient samples (2016 (*n* = 81, circle, **a**–**c**) and 2017 (*n* = 110, square, **d**–**f**)) using the trend line equations from Fig. 4d–f. Pearson’s correlation coefficient *(R)*^2^ and the corresponding *P* value (two-sided) are indicated. **g** Receiver-operating characteristics curve (ROC) analysis using samples from the 2 study years (*n* = 191 independent patient samples). Source data are provided as a Source Data file.

Given the strong correlation between the biomarker expression and a wide range of GCRs in vitro, we extended our analysis to validate the GCR prediction. To do this, we used RNA from all the samples that were assessed in our ex vivo system on D8 over two malaria seasons (Supplementary Fig. 7a)^16^. First, to independently confirm the GCRs determined microscopically in the ex vivo samples, the correlations between the microscopically defined GCR and the RNA levels of gametocyte-specific genes, *pfs25* and *pfs230*, in the ex vivo cultures on D8, were tested (Supplementary Fig. 7b) and found to be significant. The ex vivo GCRs were then compared with the relative abundance of D0 gc-ring biomarkers RNA levels in the same patient samples. There was a significant correlation between the expression levels of all three gc-ring biomarker genes and the ex vivo GCR (*ap2-g* *R*^2^ = 0.687, *surfin 1*.*2* *R*^2^ = 0.614, *surfin 13*.*1* *R*^2^ = 0.614) (Supplementary Fig. 8), as well as tight co-expression of the three gc-ring biomarkers in all the D0 clinical samples (Supplementary Fig. 9). The predicted GCR was then calculated from the gc-ring biomarker D0 ΔCts using the algorithm developed for the in vitro samples (Fig. 4d–f) and compared with the observed ex vivo GCR in samples with >0.5% D0 parasitemia (2016, *n* = 81 and 2017, *n* = 110), excluding those used in the original microarray. The >0.5% D0 parasitemia cutoff was used because of the limited sensitivity of the microscopy-based ex vivo GCR assay at low parasitemias. Good correlation was observed between the samples’ predicted and observed ex vivo GCRs (Fig. 5a–f). In addition, Bland–Altman method comparison and intra-class correlation coefficient analysis show a good agreement between both methods (Supplementary Fig. 10 and Supplementary Table 1). Moreover, the receiver-operating characteristic analysis showed all three had areas under the curve between 0.805 and 0.889, suggestive of good overall accuracy (Fig. 5g). Predicted GCRs were also similar in males and females. Together, the results strongly support the ability to predict sexual conversion using the expression levels of *ap2-g*, *surfin 1.2,* and *surfin 13.1* in malaria patient’s blood samples.

**Fig. 6:**
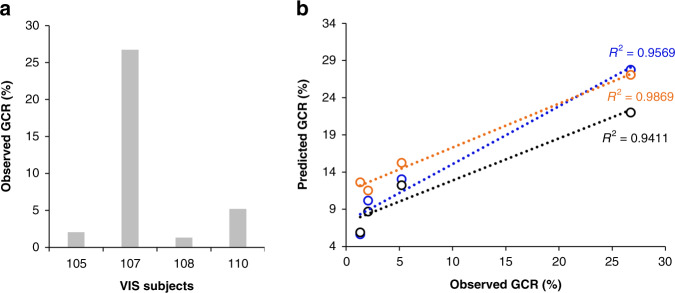
Human malaria VIS subjects’ observed and predicted GCRs correlate. Ring-stage parasite and newly released, mature gametocyte levels in four biologically independent Volunteer Infection Study (VIS) subjects (*n* = 4, 105, 107, 108, and 110) were quantified on day (D)7 and 11 days later on D18, respectively, and used to determine the observed D7-gametocyte-conversion rate (GCR) (**a**). **b** Correlation between observed and predicted D7-GCRs in VIS subjects. The predicted D7 GCR was calculated for each subject based on their D7-gametocyte-committed (gc)-ring biomarker ∆Cts and the equation developed in Fig. 4d–f. Black, blue, and orange colors indicate the predicted GCRs for the *n* = four independent subjects using *ap2-g, surfin 1.2,* and *surfin 13.1*, respectively. Source data are provided as a Source Data file.

Next, we used data from a human malaria volunteer infection study (VIS) to compare gc-ring levels with the circulation of stage V gametocytes 11 days later. The VIS study was originally conducted to test drugs for gametocytocidal efficacy^19^. Eight days after infection the participants were treated with piperaquine (PQP), an anti-malarial which only kills asexual stages allowing continued maturation and differentiation of gc-rings into mature gametocytes that were then targeted with a variety of test compounds. This experimental design provided an in vivo system to validate gc-ring biomarker GCR predictions at low parasitemias that could not be assessed using the microscopy-based ex vivo assay. The dynamics of asexual and sexual parasitemia in VIS subjects (*n* = 4) are shown in Supplementary Fig. 11. Transcript levels of the three biomarkers in RNA collected at the peak of asexual parasitemia one day prior to treatment (D7) were used to predict the number of gametocytes released into blood circulation 11 days later (i.e., D18 gametocytes minus the gametocytes already present on D17). The observed GCR was calculated by dividing the number of gametocytes released on D18 by the number of total circulating rings on D7. As in the Ghanaian malaria cases, the observed GCRs varied among the four VIS subjects (Fig. 6a) and correlated with the GCRs predicted by the biomarkers (Fig. 6b), suggesting this method could be used even at very low parasite densities (1–100 rings/µl of blood) to predict gametocyte production 11 days before the circulation of transmissible gametocytes.

### TaqMan RT-qPCR to detect gc-rings in malaria infections

**Fig. 7:**
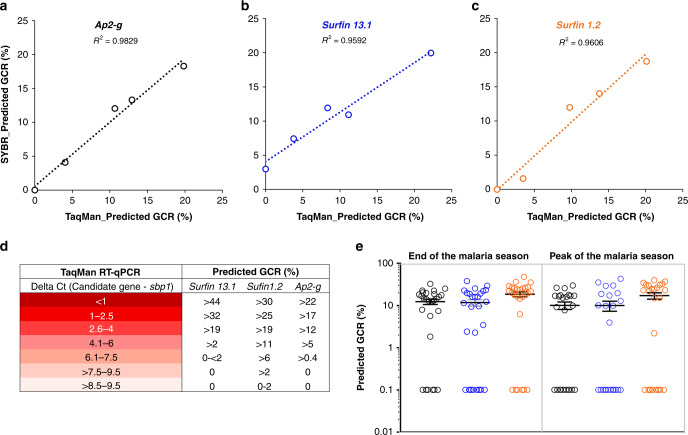
TaqMan probe-based RT-qPCR for GCR prediction. **a**–**c** Correlation between predicted gametocyte-conversion rates (GCR) using SYBR green and TaqMan RT-qPCR. Parasite lines used in this comparison were NF54, 683, 565, and *Pfgdv1.gfp.dd* grew in the presence (+) and absence (−) of Shield 1 (Shld1) (*n* = 5 biologically independent parasite lines). The equations used to predict GCR for SYBR and TaqMan RT-qPCR are shown in Fig. 4 and Supplementary Fig. 12, respectively. **d** An index of predicted GCR using a range of TaqMan RT-qPCR ΔCt for each gametocyte-committed (gc)-ring biomarker. **e** GCRs predicted using the TaqMan RT-qPCR to quantify gc-ring biomarker RNA levels in biologically independent samples from asymptomatic malaria carriers at the end (*n* = 27) and peak (*n* = 24) of the malaria season. Predicted GCRs based on *ap2-g* (black), *surfin 13.1* (blue), and *surfin 1.2* (orange) are shown. To plot on log scale, 0.1 was added to each data point, and the mean and SEM are shown. Source data are provided as a Source Data file.

To facilitate application to larger field studies, we designed a TaqMan-based RT-qPCR to increase the sensitivity, specificity, and enable multiplexing. TaqMan-based RT-qPCR was developed for each gc-ring biomarker (Supplementary Fig. 12) and the resulting GCR predictions were strongly correlated with those from the SYBR RT-qPCR (Fig. 7a–d). We extend this TaqMan-based multiplexed RT-qPCR to analyze blood samples from Ghanaian school children with asymptomatic malaria infection during the peak (July) and end (November) of the malaria season when symptomatic malaria rises and falls, respectively^20^. All study subjects were negative for malaria infection by microscopy and rapid diagnostic tests. However, by *sbp1* RT-qPCR, 57.14% (24/42) of the children at the peak and 49.09% (27/55) at the end of the malaria season had circulating ring-stage parasites. Even at these low parasite densities (0.069–80.0 parasite/µl blood), each gc-ring biomarker predicted GCR using 100 µl packed RBCs. Among parasite carriers, the proportion of children that were positive for at least two gc-ring biomarkers was higher at the end of the season (85.15%, 23/27) than during the peak season (62.50%, 15/24). The lowest predicted GCRs in these low-density asexual infections were 1.7%, 2.1%, and 2.08% for *ap2-g*, *surfin 13.1*, and *surfin 1.2*, respectively. The average GCR predicted by the ΔCt values for each biomarker was >9%, regardless of transmission season (Fig. 7e). Interestingly, a 10% GCR is the minimal level of gametocyte production required at low parasitemia (20 parasites/µL blood) to meet the theoretical transmission limit of two gametocytes/µL blood, which would be consistent with the observation that asymptomatic parasites carriers can transmit to mosquitoes^20,21^.

## Discussion

Here, we identify the genes differentially expressed in gc-ring-stage parasites circulating in malaria patient’s blood. This unique window between RBC invasion and sequestration could not be detected in vitro and provides a portal to assess malaria transmission potential prior to the release of stage V parasites into the blood circulation, which occurs ~8–12 days later. Three genes upregulated during this period were developed as gc-ring biomarkers that predict gametocyte conversion using in vitro, ex vivo, and in vivo systems. These gc-ring biomarkers can be widely used in submicroscopic to symptomatic malaria infections to detect gametocyte production in human infections in the field, as an aid to reducing malaria transmission, one of the priorities of the current research agenda^22^.

These new biomarkers allow, for the first time, the detection of immature gametocytes over a week before they can be transmitted to a mosquito, allowing the prioritization of investigational antimalarials that have activity against gc-rings and the potential to block further malaria transmission. Although, not currently a problem in areas where parasites remain artemisinin sensitive, the activity of the artemisinins against gc-rings in parasites in Southeast Asia where the K13 mutant parasite is prevalent requires monitoring^8^. Previously, the programatic switch from chloroquine to sulfadoxine/pyrimethamine, which only clears asexual rings, not gc-rings, lead to an increase in gametocytes^19,23^. The biomarkers identified here also enable wide-scale screening to develop a more comprehensive understanding of the factors that modulate the transition from asexual to sexual differentiation, as well as the survival and maturation of stage V gametocytes. The identification of individuals that do and do not effectively clear gc-rings will enable the comparison of their metabolic and immune responses to define effective natural transmission-blocking mechanisms. Naturally acquired antibodies against gametocyte antigens (e.g., Pfs48/45, Pfs230, etc.) have been associated with transmission-blocking activity in the mosquito midgut (reviewed by Acquah et al.^24^), but to date no antibodies have been identified that are specific for the surface of the intraerythrocytic gametocyte. Recently, a set of antibodies against surface molecules shared by early asexual and gc-committed parasites were reported^25^. The availability of biomarkers for both gc-rings and stage V gametocytes provides a tool to further define the role of such antibodies in the field.

Evaluating the prevalence of gc-rings in different populations and regions with different transmission dynamics will also extend our understanding of the role of host parameters in the modulation of gametocytogenesis in various malaria settings such as high- and low-endemicity, residual and active transmission seasons, and symptomatic and asymptomatic infections^16,22,26,27^. GCR variability was found in both uncomplicated malaria patients^16^ and in previously naive VIS subjects. Such variability is not surprising and has been associated with a number of factors (e.g., asexual parasitemia, fever, *gdv1* alleles, and plasma lysophosphatidylcholine levels) that could influence sexual conversion in the field^16^. In contrast, asymptomatic children had relatively high GCRs, either at the peak or end of the malaria season, which is intriguing and suggests that parasites in submicroscopic infections in the absence of symptoms produce gametocytes at a higher rate, which would increase the chance of crossing the theoretical transmission threshold (two gametocyte/µL). Individuals with submicroscopic infection, including asymptomatic or VIS subjects, can successfully transmit to mosquitoes^19–21^ consistent with the production of an adequate number of gametocytes and provides support for the high predicted GCRs in asymptomatic individuals and some VIS subjects. If confirmed by further longitudinal studies, these results suggest treatment of asymptomatic infections will be needed to advance malaria elimination efforts. Further longitudinal testing is also needed to determine whether GCR is a stable or transient characteristic, as our current work has only determined GCR at a single time point per subject.

The identification of gc-ring biomarkers could also facilitate efficacy testing of lead compounds against gametocytes, which has been challenging due to the lack of an established marker to predict gametocyte production early in the assay^28^. Previously developed transgenic reporter lines express fluorescent proteins under the *alpha tubulin II*^29^ or *gexp2*^30^ promoters to identify stage I gametocytes (~ ≥ 36 h post invasion). While these reporter lines are good for in vitro assays, neither were found to be differentially expressed in the H-GCR samples suggesting that they are only expressed after sequestration, which will limit their in vivo use in clinical trials. The gc-ring biomarkers developed in this study allow quantification of GCR before and after treatment in vitro, ex vivo or in vivo to evaluate the efficacy and stage specificity of the drug/molecule^19,23^. Additionally, gc-ring biomarker could be used to test the hypothesis that drug-resistant strains can make gc-rings at a higher rate^31^ and enhance the spread of drug resistance^8^. Identifying this tendency early in the drug development process will allow for appropriate surveillance and management strategies to be put into place. As a note, gc-ring biomarkers are unable to distinguish male and female gametocytes, and the relationship between sex ratio and conversion rates needs to be explored in future studies, as earlier work demonstrates the importance of this to design interventions^32^.

The AP2-G-dependence of all the genes differentially overexpressed in the H-GCR cohort provides a separate line of support for their gc-ring specific upregulation. In addition to monitoring AP2-G dependence by treating the AP2-G inducible line (*E5.ap2-g.dd*) with a single Shld1 concentration in triplicate (Fig. 2a), the dose-response experiment independently tested gc-ring gene expression at 6 different Shld1 concentrations in duplicate (Fig. 2c) increasing confidence in the results. Additionally, AP2-G-dependent regulation of the transcript levels of 10 of the 18 genes was also corroborated in vitro^33^. However, five of the 18 gc-ring genes have not been identified in prior in vitro studies, including the two *surfins* selected as gc-ring biomarkers. The *surfins* are members of a multigene family characterized by the presence of a schizont-infected cell agglutination (SICA) C-terminal inner membrane domain^34^. This domain was first identified in *P. knowlesi* and thought to be exposed on the RBC surface. The *surfins* also have a noncanonical export signal^35^, consistent with transport to the RBC and, possibly, exposure on the iRBC surface. Together, these characteristics suggest that SURFIN 1.2 and 13.1 could be involved in modifications of the gc-ring-infected erythrocyte that may provide the first step toward sequestration; however, this needs to be investigated further. It is not clear why these genes were not identified in previous work. It could be due to their relatively short duration of expression, challenges associated with experimentally synchronizing and regulating gametocyte production, or the low yield of RNA from ring-stage parasites. It is also possible that the gene expression levels are higher in the parasite’s natural niche than in culture^36^.

While gc-ring biomarkers offer exciting tools to enhance malaria elimination by identifying transmission hotspots and determinants of the sexual conversion, to date they have only been studied in field samples obtained in the central region of Ghana over the course of 2 years and in lab-adapted parasite lines. Additional studies using samples collected from diverse malaria settings are needed to ensure their general applicability. More information is also needed about the relationship between gc-rings and sequestered immature gametocytes and whether gc-ring biomarkers can predict the sequestered reservoir of immature gametocytes. The algorithm we have developed is based on a range of in vitro GCRs which varies from 0 to 20.85% and we have observed a decrease in sensitivity of GCR prediction using gc-ring biomarkers if the observed GCR is at or beyond the limits of this range (<1% and >23%).

The gametocyte prognosis tools developed here will sharpen the existing molecular arsenal targeting malaria elimination. This early detection provides an 8–12 day window for treatment prior to transmission and can be used with submicroscopic and symptomatic infections. Additionally, these gc-ring biomarkers are vital to investigating host and parasite factors that drive sexual conversion and modulate gametocyte survival in the field. They also expedite the evaluation of drug efficacy against sexual stages.

## Methods

### Ethical statements

The clinical study undertaken in Ghana was approved by the Institutional Review Board of the Noguchi Memorial Institute for Medical Research and the Ghana Health Services and reviewed by DMID, NIAID (NIH). After informing parents/guardians about the study’s objectives, methods, anticipated benefits, and potential hazards, study subjects were recruited. The parents/guardians were encouraged to ask questions about any aspect of the study that was unclear to them and informed about their liberty to withdraw their children at any time without penalty. Children were enrolled only after written parental consent had been obtained. All patient information was treated as confidential. The Volunteer Infection Study (VIS) was reviewed and approved by the QIMR Berghofer Medical Research Institute Human Research Ethics Committee, and all participants gave written informed consent before inclusion in the study. The study was registered with ClinicalTrials.gov (NCT02431650), and has been reported previously^19^. The current exploratory study was approved by the QIMR Berghofer Medical Research Institute Human Research Ethics Committee reference number P3498. The inclusion of VIS participants in this study was based on the consent for future research and the availability of RNA material.

### Study sites and asymptomatic malaria infection survey

Blood samples of children (≤13 yrs) with symptomatic malaria, were collected at the Ewim Health Center in Cape Coast, Ghana, as part of a study evaluating ex vivo gametocyte production^16^. In brief, parental consent was obtained for children (≤13 yrs) with *P. falciparum* parasitemia ranging between 1000 and 250,000 per µl of blood to donate 5 ml of blood to evaluate hemoglobin levels, white blood cell count, and plasma components as well as the parasite population. The child’s age, sex, and axillary temperature were also recorded. For the asymptomatic malaria study, children (5 to ≤13 yrs) attending schools at Simiw (Komenda Edina Eguafu Abirem District in the Central Region of Ghana) were recruited. The survey was conducted at the end (November 2017) and peak (July 2018) of the malaria transmission seasons. During both surveys, children were asymptomatic (afebrile with no other malaria-related symptoms). One milliliter of venous blood was drawn from each subject and an aliquot used to prepare blood smears and spot HRP2 based malaria RDTs. Subsequently, plasma was separated and stored at −80 °C. In total, 100 µl packed RBCs, which is equivalent to ~250 µl whole blood (depending on the hematocrit), were preserved in NucleoZOL for RNA analysis.

### In vitro *P. falciparum* culture and ex vivo gametocyte assay

All parasite strains (NF54, *E5.ap2-g.dd*^17^, *Pfgdv1.gfp.dd*^16^) were cultured in a complete RPMI medium containing RPMI 1640, 25 mM HEPES, 100 μg ml^−1^ of hypoxanthine, and 0.3 mg ml^−1^ of glutamine (KD Biomedical, Columbia, MD) supplemented with 25 mM NaHCO_3_ (pH 7.3), 5 μg ml^−1^ of gentamicin, and 10% human serum (Interstate Blood Bank, Memphis, TN). Transformed lines were maintained in 2.5 nM WR99210 (WR) (Jacobus Pharmaceuticals, Plainsboro, NJ). Sorbitol treatment (5%, 10–30 min at 37 °C) was used for synchronizing parasites at the ring-stage. Parasitemia was evaluated through microscopy after Giemsa staining smears. Parasite clones isolated from Ghanaian clinical samples (592, 607, 565, and 683) were grown in complete RPMI medium as described except 10% human serum which was substituted with 0.5% Albumax-II+2% human serum. In vitro and ex vivo gametocyte assays were described previously in full detail^16^ and used to define the cutoffs for H- and L-GCR as >2% and <0.1%, respectively.

### Whole-genome-expression profiling

An Agilent microarray containing probes for 5254 *P. falciparum* 3D7 transcripts arrayed in 15,000 spots^17^ was probed with RNA isolated from the 2016 H-GCR and the L-GCR whole-blood samples at the Johns Hopkins core facility [http://www.microarray.jhmi.edu/], and the normalized data were sent to USU for further analysis. False discovery rate (FDR) was calculated to control for multiple comparisons using the *q*-value method (FDR *q*-value). A Morpheus web-based tool [https://software.broadinstitute.org/morpheus/] was used to generate the heatmap and to determine the hierarchical clusters of the blood samples based on the expression profile of 29 genes expressed differentially (*n* = 18) or similarly (*n* = 11) between H and L-GCR groups. The clustering pattern remained consistent when generated using only differentially expressed or using all 29 genes. Euclidean distance and average linkages between all pairs of samples were used to determine cluster distances.

### RNA extraction and RT-qPCR

RNA was extracted from parasites preserved in NucleoZOL (MACHEREY-NAGEL, USA) or Paxgene tubes (BD Biosciences) using RNeasy Micro kit (Qiagen, Germany) or Paxgene RNA kit (Qiagen, Germany), respectively, as per the manufacturer’s instructions. RNA was eluted in 80 µl and 36 µl RNAse-free water from Paxgene RNA kit and RNeasy Micro kit, respectively. Purified RNA (8.0 µl) was additionally treated with ezDNAse™ before cDNA synthesis using SuperScript™ IV VILO™ Master Mix (Invitrogen, USA). 1:10 dilution of cDNA was made and 2 µl from this dilution was used for each RT-qPCR well. RT-qPCR (QuantStudio3) was performed to quantify transcript levels using gene-specific primers and probes (Supplementary Tables 2 and 3). Thermal cycling conditions for Fast SYBR Green PCR Master Mix were described earlier^16^, whereas the TaqMan™ fast advanced master mix (Applied Biosystems, USA) thermal cycling conditions were 20 s activation at 95 °C, 40 cycles of 1 s at 95 °C and 20 s at 60 °C. All samples were run in duplicate and tested for both the gene of interest and the control constitutive gene, *pf18s rRNA* or *sbp1*, on the same plate. These data were analyzed using QuantStudio™ Design & Analysis Software v1.3.1, and the ΔCt values were determined by subtracting the mean cycle threshold (Ct) value for *pf18s rRNA* or *sbp1* from the mean Ct of each test gene. The relative quantity (2^−ΔΔCt^) was calculated for each gene using RNA from *NF54gdv1.gfp.dd* parasites were grown in the absence of Shld1, as described earlier^16^. Primers were designed using the web-based tool Primer3Plus. DNA sequence of each gene was downloaded from the Plasmodium genome database (PlasmoDB)^37^. Primer efficiency was tested by serial dilution and ranged from 85 to 105%.

### The Volunteer Infection Study

Details of the study design and participants were published previously along with the primary and secondary outcomes^19^. In brief, four subjects enrolled in the OZGAM open-label clinical trial were selected for the current study based on the availability of RNA samples. Malaria-naive adults were inoculated with ~2800 *P. falciparum* 3D7 parasites. Total parasitemia and gametocytemia were evaluated using *pf18s rRNA* qPCR and *pfs25* RT-qPCR, respectively. Two of these subjects received OZGAM (500 mg; formerly known as OZ439) on day 25 post-inoculation and the other two received Primaquine, 15 mg on day 24 and 45 mg on day 29 post-inoculation. In this study, only parasite RNA samples collected at different time points were used to validate GCR prediction of gc-ring biomarkers.

### Statistical analysis

All statistical analyses were performed using GraphPad Prism 7.00 statistical package^38^, except Intra-class correlation coefficient (ICC) and receiver-operating characteristics (ROC) curve analysis which were done using SPSS v24^39^. All comparisons between H- and L-GCR groups were analyzed using a nonparametric unpaired *t* test (Mann–Whitney). Pearson correlation coefficient was analyzed using Microsoft excel.

### Reporting summary

Further information on research design is available in the Nature Research Reporting Summary linked to this article.

## Supplementary information

Supplementary file

Reporting Summary

## Data Availability

All data associated with this study are present in the paper or the Supplementary Materials. Microarray data were submitted to Gene Expression Omnibus (GEO) public repository and data can be accessed through accession number GSE152536. Source data are provided with this paper.

## Source data

Source data
