# Author Correction: The transcriptome of circulating sexually committed *Plasmodium falciparum* ring stage parasites forecasts malaria transmission potential

**DOI:** 10.1038/s41467-022-30830-6

**Published:** 2022-05-30

**Authors:** Surendra K. Prajapati, Ruth Ayanful-Torgby, Zuleima Pava, Michelle C. Barbeau, Festus K. Acquah, Elizabeth Cudjoe, Courage Kakaney, Jones A. Amponsah, Evans Obboh, Anwar E. Ahmed, Benjamin K. Abuaku, James S. McCarthy, Linda E. Amoah, Kim C. Williamson

**Affiliations:** 1 Department of Microbiology and Immunology, Uniformed Services University of the Health Sciences, Bethesda, MD USA; 2 Noguchi Memorial Institute for Medical Research, University of Ghana, Accra, Ghana; 3 QIMR Berghofer Medical Research Institute, Brisbane, QLD Australia; 4 University of Cape Coast, Cape Coast, Ghana; 5 Department of Preventive Medicine and Biostatistics, Uniformed Services University of the Health Sciences, Bethesda, MD USA; 6 Present Address: University of Virginia, Charlottesville, VA USA

**Keywords:** Parasite biology, Parasite genomics, Biomarkers, Prognostic markers

Correction to: *Nature Communications* 10.1038/s41467-020-19988-z, published online 02 December 2020.

The original version of this Article contained an error in Supplementary Table 3 giving details on used primers and probes. The combination of probe and primer pair ‘Ap2g_TaqMan_1F’ and ‘Ap2g_TaqMan_1R’ and associated sequences for Ap2-g were incorrect. This has been corrected to primer pair ‘Ap2g_TaqMan_2F’ and ‘Ap2g_TaqMan_2R’ and their respective sequences in Supplementary Table 3. This error has been corrected in the HTML versions of the Article.

## Supplementary information

Updated Supplementary Information

