# Supplementary material for: The transcriptome of circulating sexually committed Plasmodium falciparum ring stage parasites forecasts malaria transmission potential
Source: Nat Commun. 2020 Dec 2;11:6159. doi: 10.1038/s41467-020-19988-z (PMC7710746; doi:10.1038/s41467-020-19988-z)
Supplement: Supplementary file 1 — Supplementary file [file 41467_2020_19988_MOESM1_ESM.pdf]

Supplementary materials

The transcriptome of circulating sexually committed *Plasmodium falciparum* ring stage parasites forecasts malaria transmission potential

Surendra K. Prajapati[1*], Ruth Ayanful-Torgby[2], Zuleima Pava[3], Michelle C. Barbeau[1†], Festus K. Acquah[2], Elizabeth Cudjoe[2], Courage Kakaney[2], Jones A. Amponsah[2], Evans Obboh[4], Anwar E. Ahmed[5], Benjamin K. Abuaku[2], James S. McCarthy[3], Linda E. Amoah[2], Kim C. Williamson[1]
[1]Department of Microbiology and Immunology, Uniformed Services University of the Health Sciences, Bethesda, Maryland, USA. [2]Noguchi Memorial Institute for Medical Research, University of Ghana, Accra, Ghana, [3]QIMR Berghofer Medical Research Institute, Brisbane, Australia, [4]University of Cape Coast, Cape Coast, Ghana, [5]Department of Preventive Medicine and Biostatistics, Uniformed Services University of the Health Sciences, Bethesda, Maryland, USA. †Current address (University of Virginia, Charlottesville, Virginia, USA), *Correspondence and requests for materials should be addressed to (surendra.prajapati.ctr@usuhs.edu)

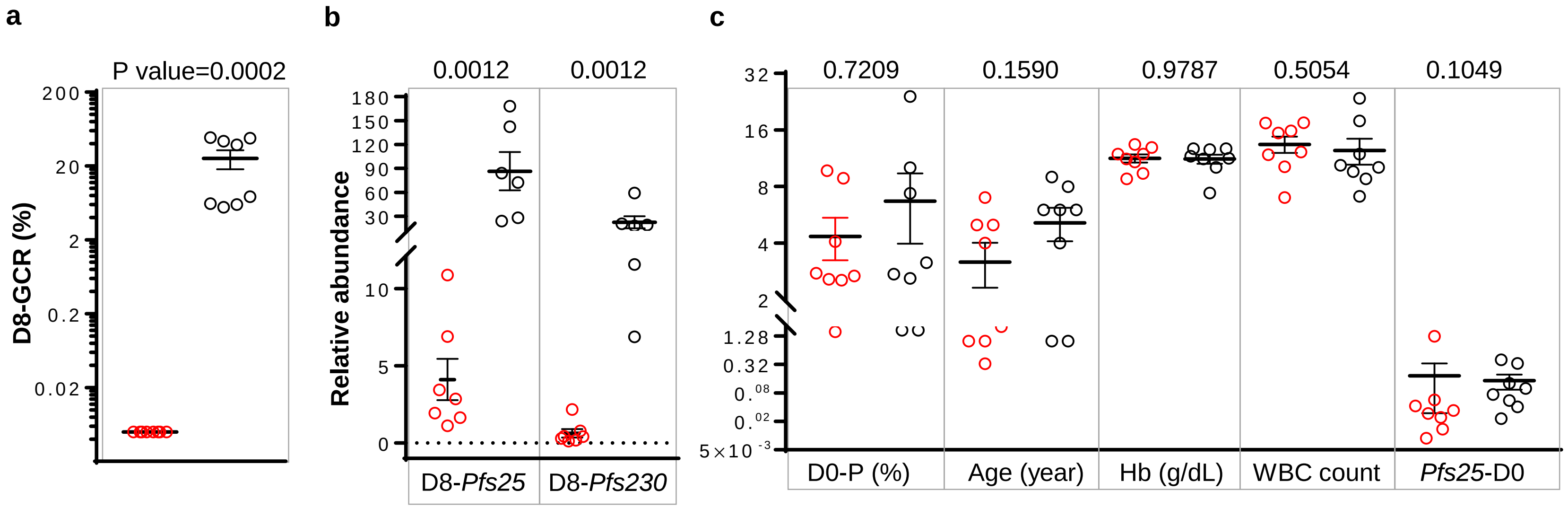

Supplementary Fig. 1. High (H)- and low (L)-gametocyte conversion rate (GCR) samples and patient parameters. a) The Day(D)8 GCR for the biologically independent samples in the H- (black circle, n=8) and L-GCR (red circle, n=8) cohorts. **b**) Transcript abundance of *pfs25* and *pfs230* in the same biologically independent D8 ex vivo samples confirms the H-(n=8) and L(n=8)-GCR groups defined by microscopy. **c**) The clinical parameters of the H- and L-GCR patients (n=8 each group) that contributed the samples assessed in **a&b**, including D0 parasitemia (%), age, hemoglobin levels, WBC counts and transcript abundance of *pfs25* in D0 samples, were randomly distributed in both groups. **a-c**) The mean and standard error of the mean (SEM) are shown. **a-c)** Differences between the H- and L-GCR were evaluated using a two-sided Mann Whitney test and the P values indicated. Source data are provided as a Source Data file.

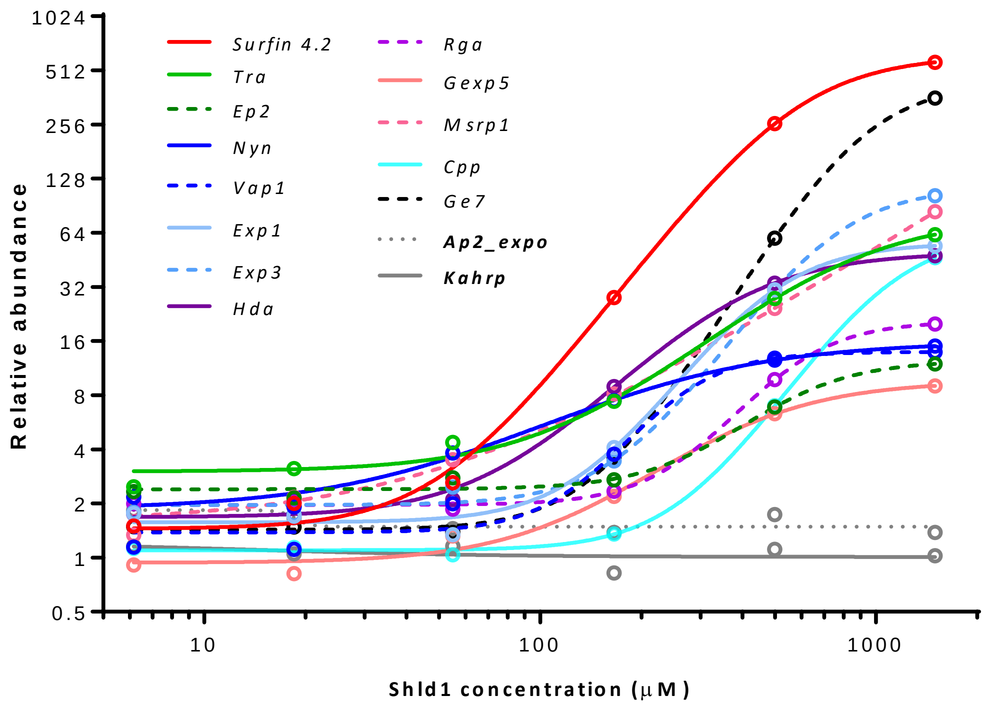

Supplementary Fig. 2. AP2-G augments expression of high (H)-GCR associated genes. The fold change in gene expression between parasites treated with or without the indicated concentration of Shield 1 (Shld1). *Kahrp* and *ap2_expo* indicated in bold where included as asexual controls. The mean from n = 2 independent experiments at each Shld1 concentration is shown. Source data are provided as a Source Data file.

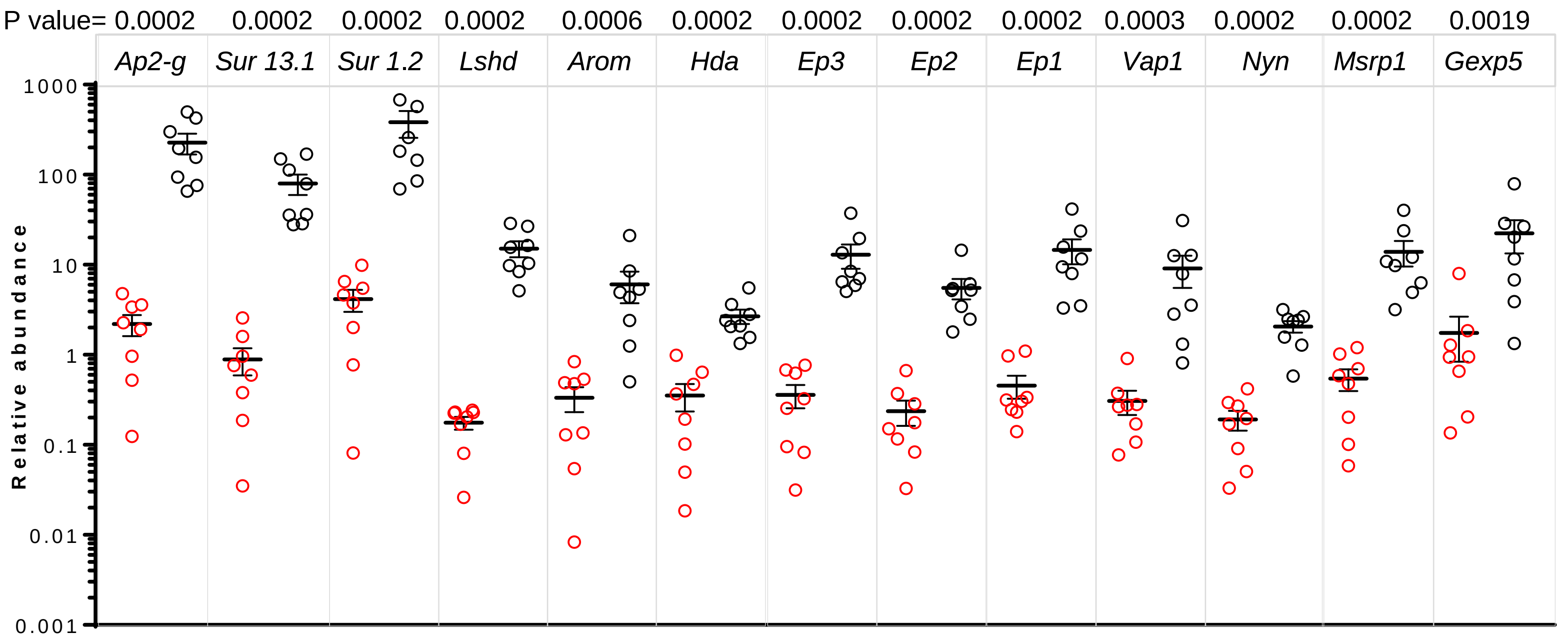

Supplementary Fig. 3. RT-qPCR validation of differential expression in the high (H)-GCR D0 RNA samples. Relative abundance of 13 genes determined by RT-qPCR using the same biologically independent H- (black circles, n=8) and low (L)- (red circles, n=8) gametocyte conversion rate (GCR) samples analyzed by microarray. The mean and standard error of the mean (SEM) are shown for both groups. Differences between the H- and L-GCR were evaluated using a two-sided Mann Whitney test and the P values indicated. Source data are provided as a Source Data file.

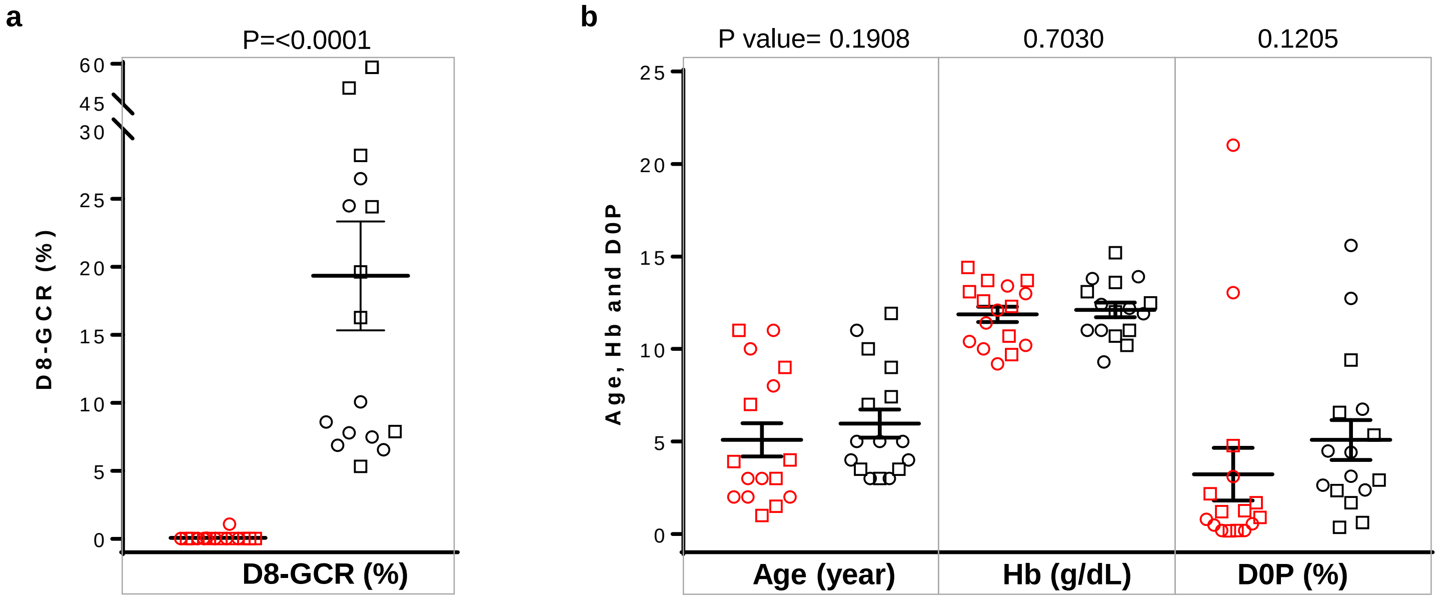

Supplementary Fig. 4. Characteristics of additional high (H)- and low (L)-GCR cohorts from 2016 and 2017. a) Distribution of Day(D)8- gametocyte conversion rate (GCR) in additional biologically independent samples selected from the 2016 (circle) and 2017 (squares) H- (n=16, black symbol) and L-GCR (n=16, red symbol) cohorts. **b**) The clinical parameters of the H- and L-GCR patients (n=16 each group) that contributed the samples assessed in **a,** including age, hemoglobin levels and D0 parasitemia (%). The mean and standard error of the mean (SEM) of the groups is shown and differences between the H- and L-GCR cohorts were evaluated using a two-sided Mann Whitney test. The P values are indicated. Source data are provided as a Source Data file.

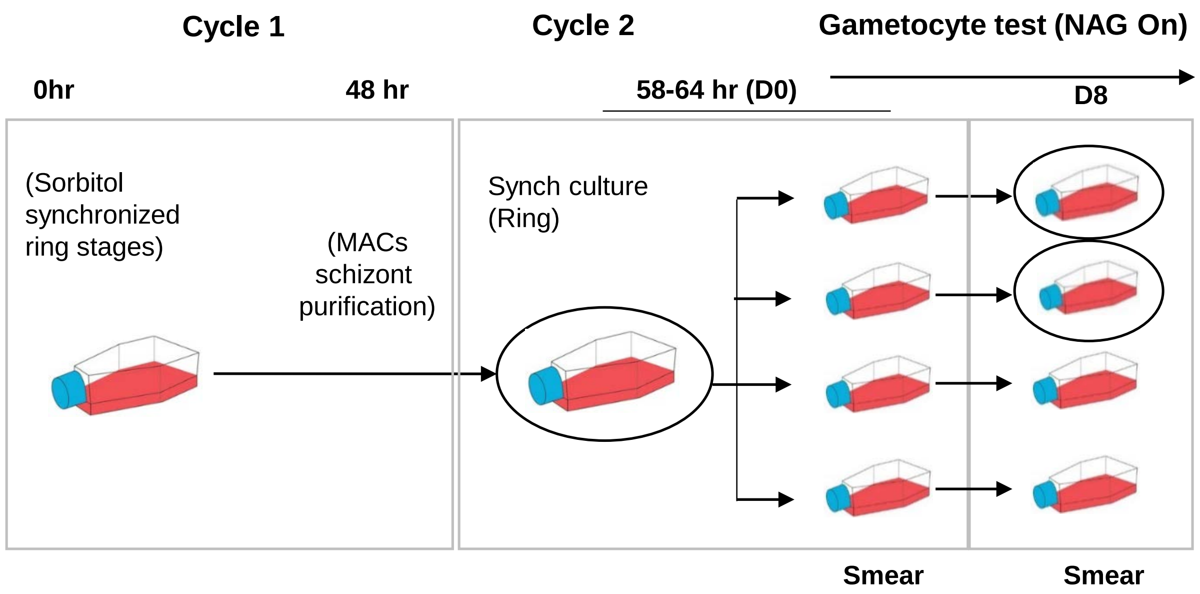

Supplementary Fig. 5. Experimental outline of in vitro gametocyte assay. Sorbitol synchronized ring stage cultures were allowed to develop into mature schizonts (44-48 hour post invasion) before MACS purification. The purified schizonts were returned to culture and 12 hours later aliquots were removed for RNA isolation (circle) and a Giemsa-stained smear. N-acetyl glucosamine (NAG) was added to the rest of the culture and it was split into 4 flasks. Two flasks were harvested for RNA isolation (circle) and a Giemsa-stained smear on Day(D)8.

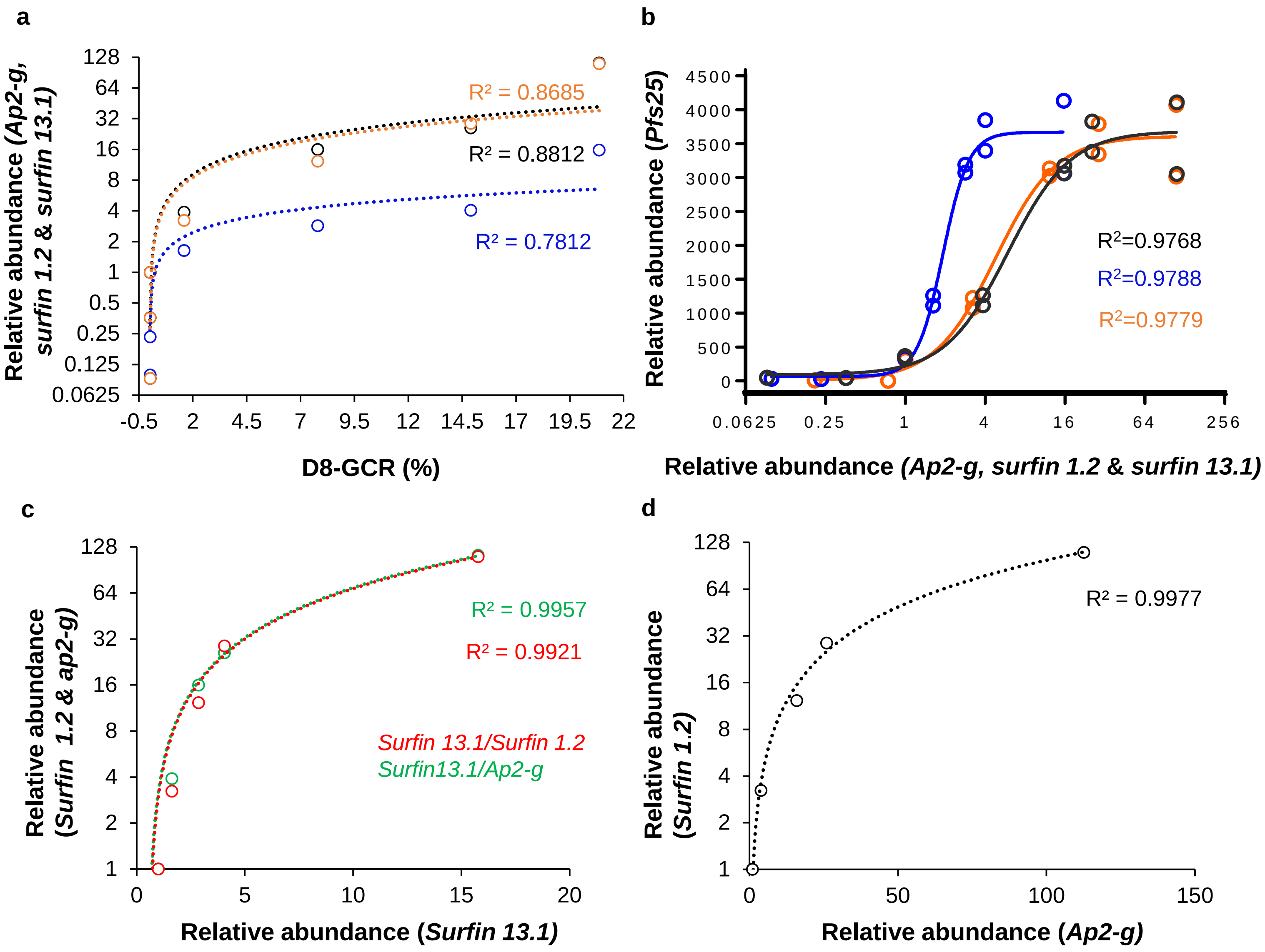

Supplementary Fig. 6. GCR and gametocyte committed (gc)-ring biomarkers expression level correlations in in vitro samples. Transcript abundance of *ap2-g* (black), *surfin 13.1* (blue) and *surfin 1.2* (orange) in Day(D)0 RNA from the n = 7 independent parasite lines with different conversion rates described in Fig. 4 are plotted against D8 gametocyte conversion rates (GCRs) determined microscopically (**a**) and by *pfs25* RNA levels (**b**). Transcript abundance of all three genes for n= 5 independent parasite lines, all 4 H GCR lines (683, 565, NF54, and Pfgdv1.gfp.dd +Shld) and the reference L-GCR line, (Pfgdv1.gfp.dd -Shld1) were compared with each other (**c-d**). The R^2 values are indicated. Source data are provided as a Source Data file.

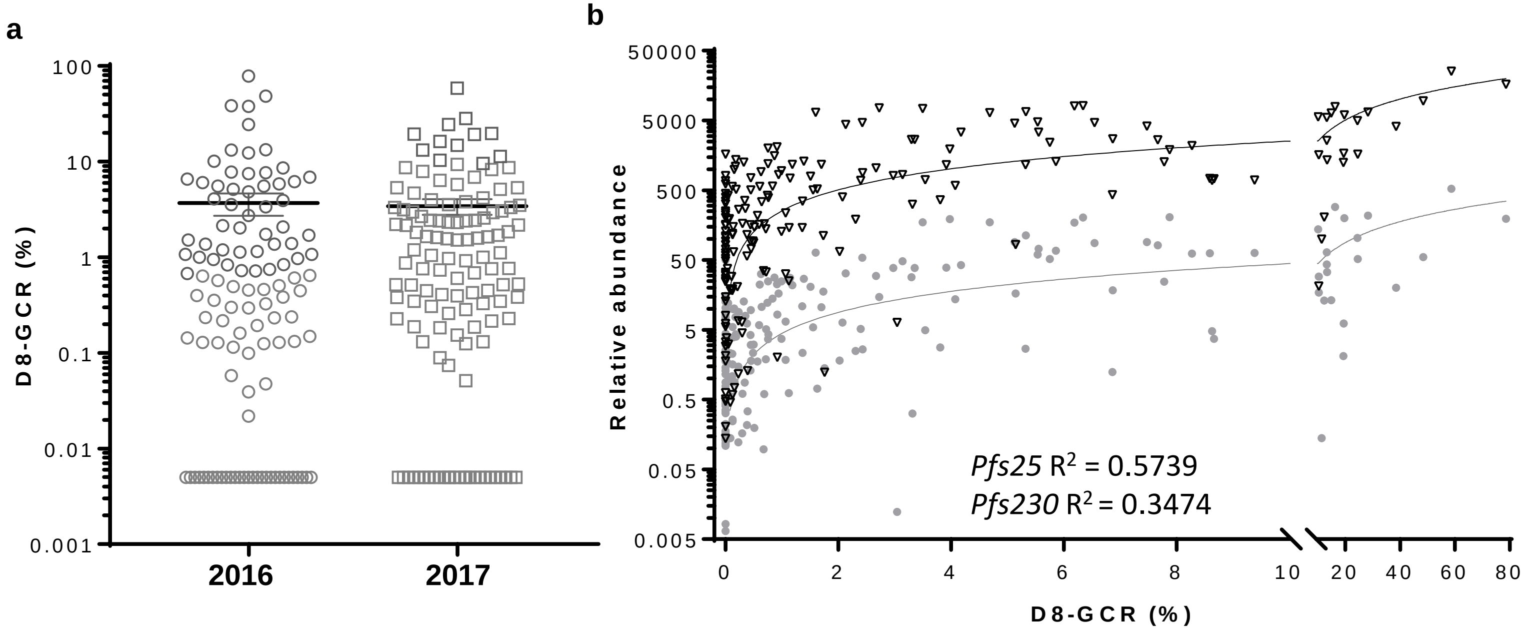

Supplementary Fig. 7. Molecular validation of the ex vivo D8 GCR determined by microscopy. a) Day(D)8 ex vivo gametocyte conversion rates (GCRs) determined microscopically as described by Usui et al 2019 for biologically independent samples collected in 2016 (n=113, circles) and 2017 (n=125, squares). To graph this on a log plot, 0.005 was added to each sample. The mean and standard error of the mean (SEM) for both groups is indicated. **b**) The relative abundance of two mature gametocyte-specific genes, *pfs230* (filled circle) and *pfs25,* (open triangle) in RNA collected on ex vivo day (D)8 from the same set of biologically independent samples used in **(a)** [2016 (n=112) and 2017 (n=88)] are plotted against the microscopically determined D8-GCRs. The trend line and R^2 value are indicated. Source data are provided as a Source Data file.

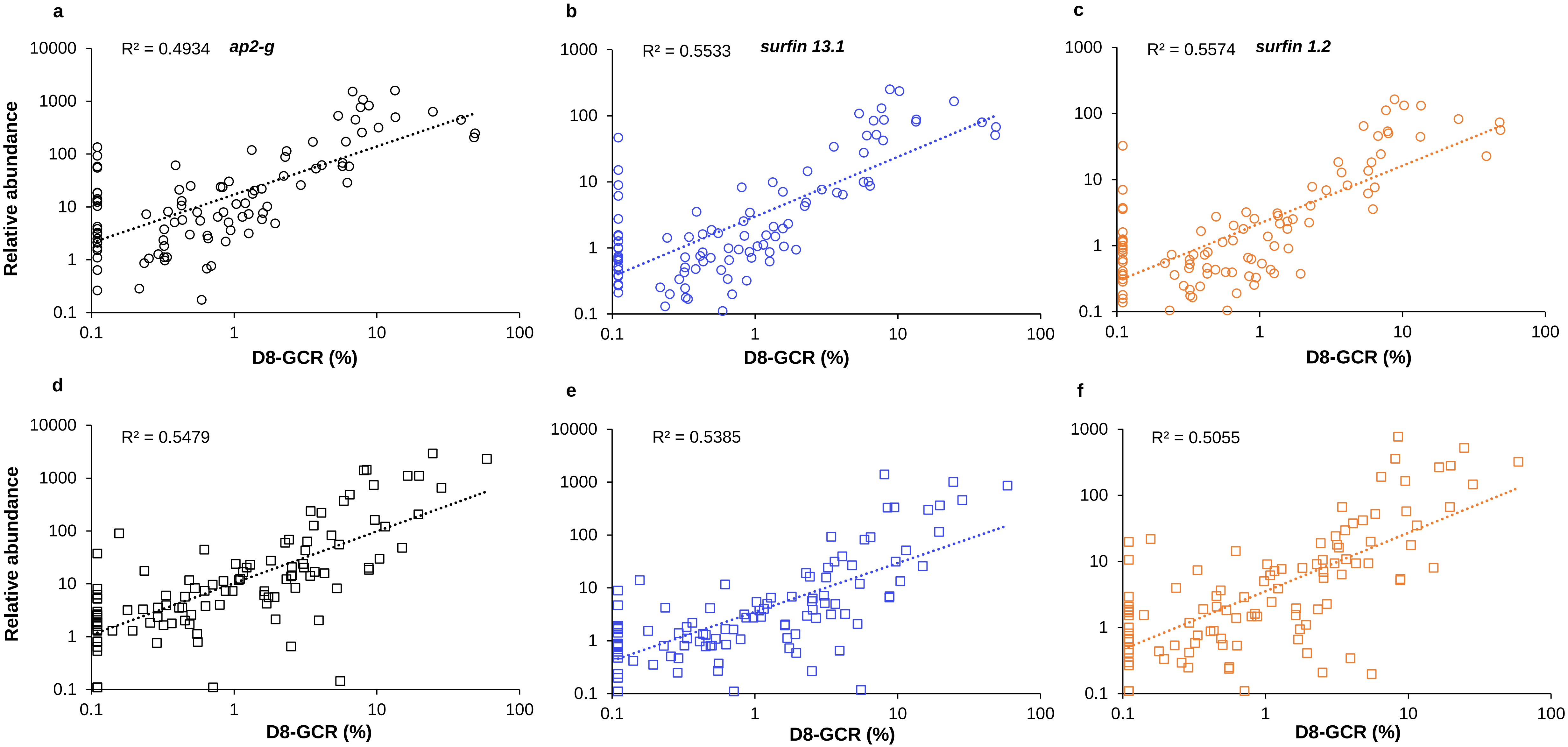

Supplementary Fig. 8. Ex vivo D8-gametocyte conversion rates (GCRs) correlate with the transcript abundance of gametocyte committed (gc)-ring biomarkers. The transcript abundance of *ap2-g* (black, **a** & **d**), *surfin 13.1* (blue, **b** & **e**) and *surfin 1.2* (orange, **c** & **f**) in Day(D)0 RNA from biologically independent samples collected in 2016 (circle, **a-c**, n=96) and 2017 (square, **d-f**, n=110). The trend line and R^2 value are indicated. Source data are provided as a Source Data file.

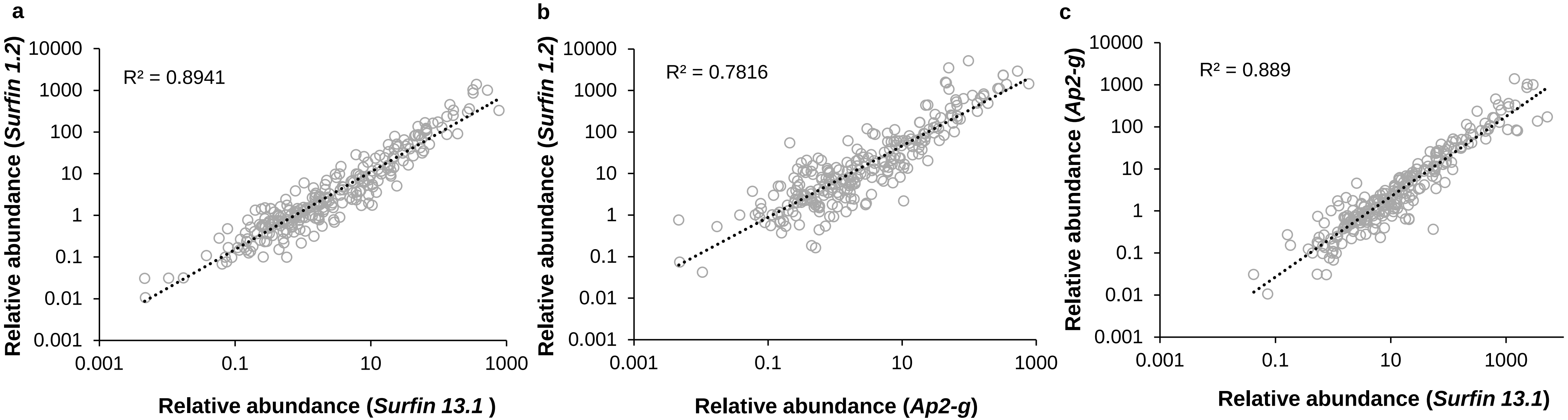

Supplementary Fig. 9. Correlation between the transcript abundance of all three gametocyte committed (gc)-ring biomarkers in the Day(D)0 samples from 2016 and 2017. The relative abundance of *surfin 1.2* was compared with the relative abundance of *surfin 13.1* (**a**) and *ap2-g* (**b**) in all the biologically independent samples [year 2016 (n=123) and year 2017 (n=134)], as were the abundance of *ap2-g* and *surfin 13.1* (**c**). The trend line and R^2 value are indicated. Source data are provided as a Source Data file.

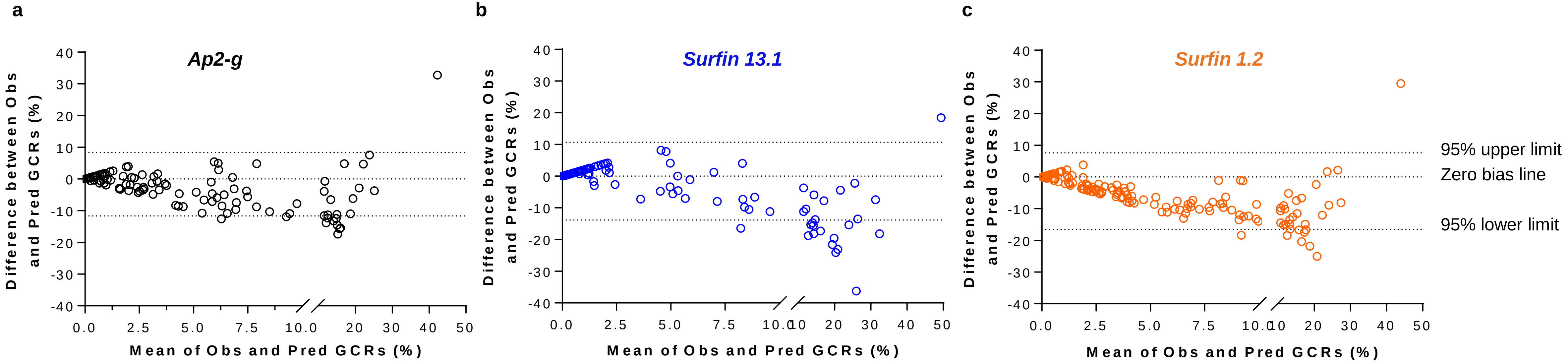

Supplementary Fig. 10. Bland-Altman method comparison of D8-observed and predicted GCRs. This comparison has included all biologically independent samples from 2016 (n=81) and 2017 (n=110), except those included in the original microarray. Black (**a**), blue (**b**) and orange (**c**) colors indicate *ap2-g, surfin 13.1* and *surfin 1.2*, respectively. Obs indicates observed Day(D)8-ex vivo gametocyte conversion rate (GCR) and Pred indicates for gametocyte committed (gc)-ring biomarkers predicted GCR. Source data are provided as a Source Data file.

Supplementary Table 1. Inter-rater agreement between observed D8-GCR and predicted GCR

**Measurements**	**ICC**	**95% CI for ICC**	**Agreement**
Observed D8-GCR & *Ap2-g* predicted GCR	0.817*	0.756, 0.862	Good
Observed D8-GCR & *Surfin 13.1* predicted GCR	0.814*	0.752, 0.860	Good
Observed D8-GCR *& Sufin1.2* predicted GCR	0.782*	0.710, 0.836	Good

ICC: Intra-class correlation coefficient, CI: confidence interval, Observed: Observed D8-ex vivo GCR, Predicted: gc-ring biomarker predicted GCR. *Bonferroni's adjusted two sided P-value < 0.0167.

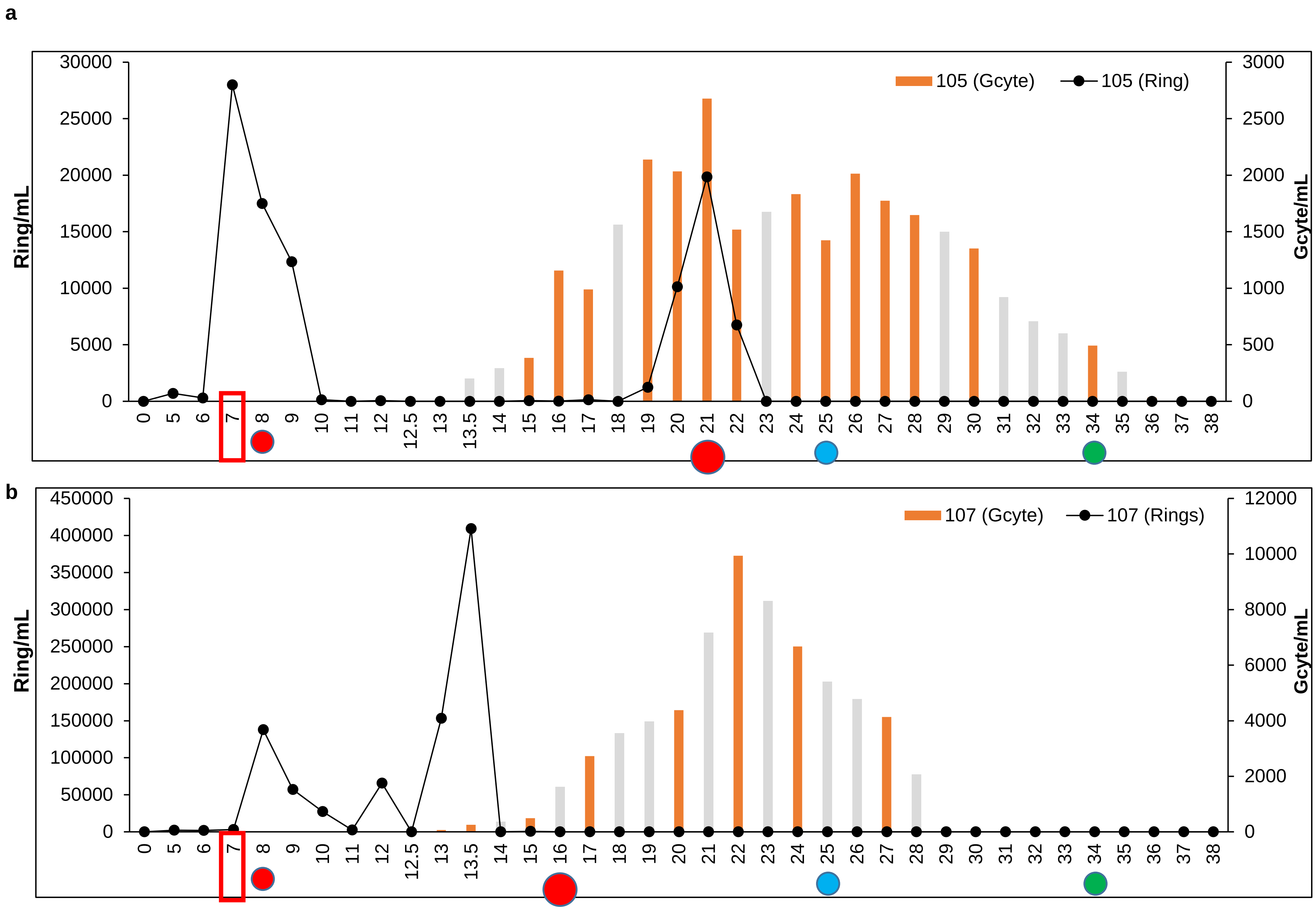

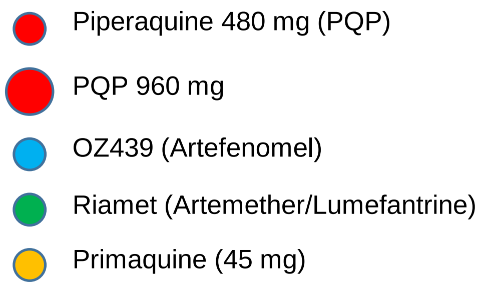

Supplementary Fig. 11. Parasite dynamics in the human malaria Volunteer Infection Study (VIS) subjects. Parasite concentrations (rings/ml, black line/left y axis and gametocytes/ml, orange columns/right y axis) are plotted over time following the initiation of a blood stage Pf VIS in 4 subjects, 105 (**a**), 107 (**b**), 108 (**c**) and 110 (**d**). Drug treatment days are shown for each subject. Ring stage parasites were quantified in subject blood samples on the indicated day using *sbp1* transcript and gametocytes were quantified using *pfs25* RNA levels. The grey columns are estimates of gametocyte for unavailable samples. Levels were estimated by taking the average of the two days flanking the missing data. Transcript levels of (*ap2-g, surfin 13.1* and *surfin 1.2*) were tested on Day(D)7 and used to predict the number of gametocytes released into circulation 11 days later on D18. Source data are provided as a Source Data file.

c

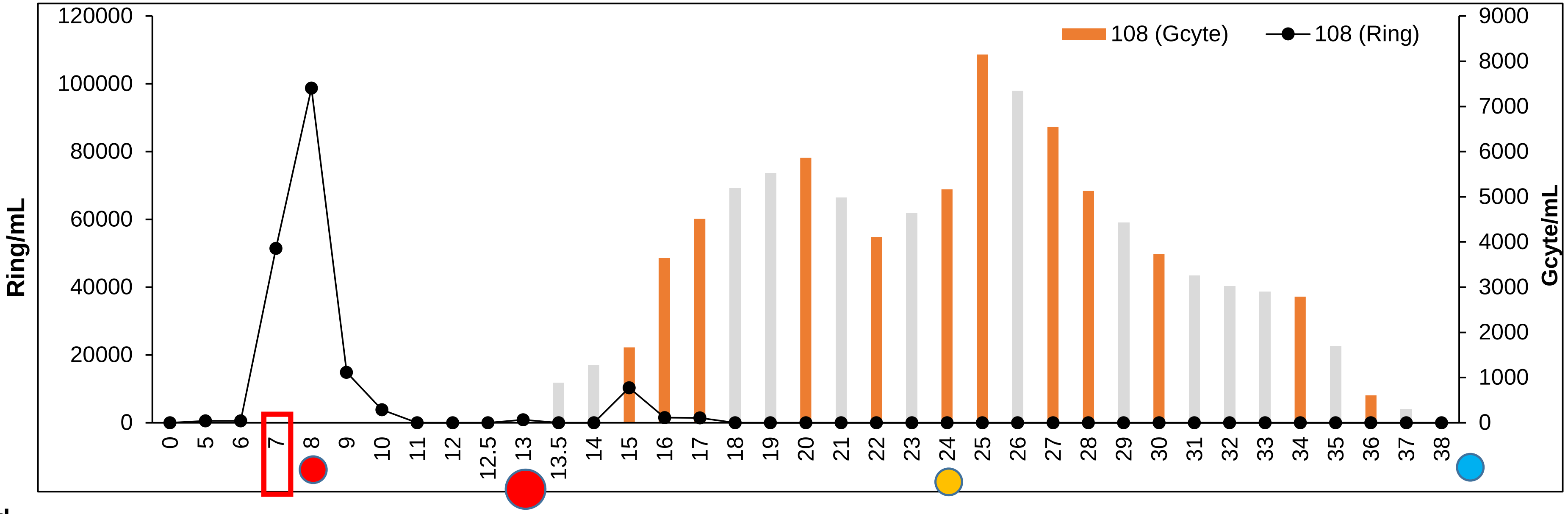
108 (Gcyte)
108 (Ring)
Ring/mL
Gcyte/mL
120000
100000
80000
60000
40000
20000
0
9000
8000
7000
6000
5000
4000
3000
2000
1000
0
0 5 6 7 8 9 10 11 12 12.5 13 13.5 14 15 16 17 18 19 20 21 22 23 24 25 26 27 28 29 30 31 32 33 34 35 36 37 38

d

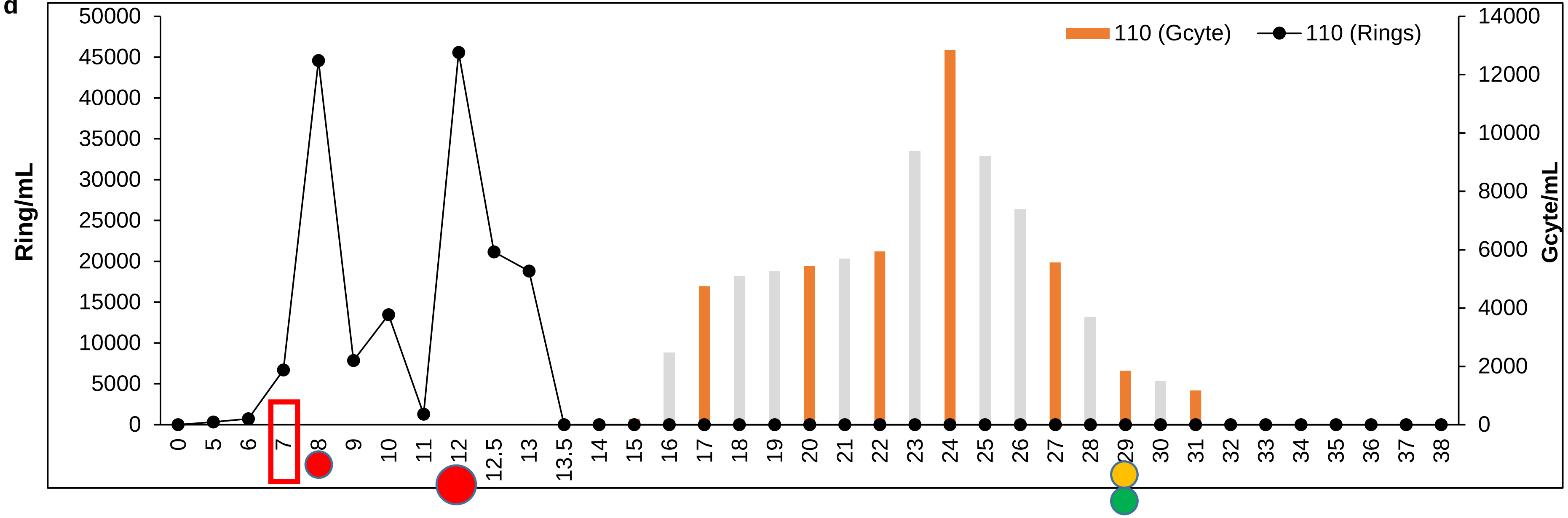
110 (Gcyte)
110 (Rings)
Ring/mL
Gcyte/mL
50000
45000
40000
35000
30000
25000
20000
15000
10000
5000
0
14000
12000
10000
8000
6000
4000
2000
0
0 5 6 7 8 9 10 11 12 12.5 13 13.5 14 15 16 17 18 19 20 21 22 23 24 25 26 27 28 29 30 31 32 33 34 35 36 37 38

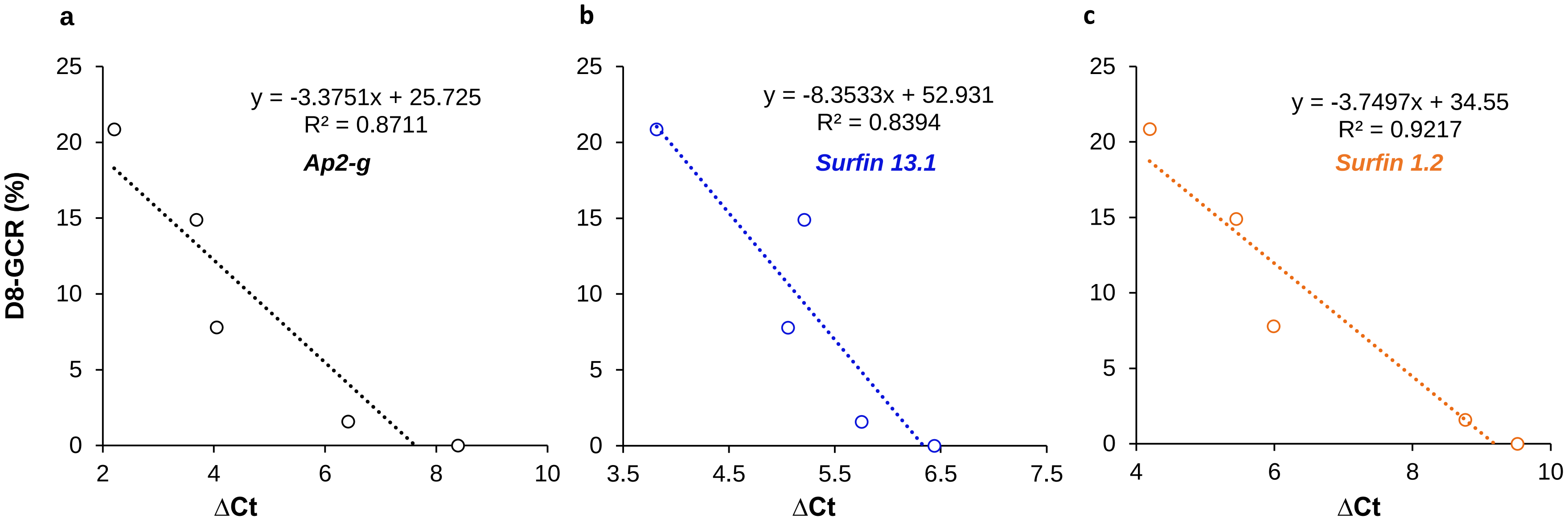

Supplementary Fig. 12. Correlation between D8-GCR and GC-ring biomarker's ΔCt determined using a TaqMan RT-qPCR. In vitro Day(D)8 gametocyte conversion rates (GCRs) obtained from gametocyte producer [NF54, 683, 565 and *Pfgdv1.gfp.dd* (+ Shld1)] and deficient [*Pfgdv1.gfp.dd* (- Shld1)] lines were plotted against the ΔCt of the indicated gametocyte committed (gc)-ring biomarker, *ap2-g* (**a, black**), *surfin 13.1* (**b**, **blue**) and *surfin 1.2* (**c**, **orange**) transcripts using *sbp1* transcript levels as the endogenous control. The linear trend line is indicated with its equation and the R^2 value. Source data are provided as a Source Data file.

Supplementary Table 2. Details of primers used in SYBR green RT-qPCR

Gene	Primer name	Primer Sequence (5'-3')	Gene	Primer name	Primer Sequence (5'-3')
Ap2-g	Ap2-g_F:	CCTGTATACCCTTCTTCGAAAGC	*Cpp*	CPP_F:	TGCTCAGGTACAAACCAACG
	Ap2-g_R:	TCAAAAGTGCTCTCCTTTCGTG		CPP_R:	TTCTACATCCGAATCGACCA
Surfin 13.1	Surfin_13.1_F	ACCCGAAGTGACAACATCTCC	*Pfge7*	Pfge7_F:	GGCCAAAATGAGGATGTTGA
	Surfin_13.1_R	TCTCCACGAGTTCCAAGTTTT		Pfge7_R:	CGCTAGTATTGGGTGAAGCA
Surfin 1.2	Surfin_1.2_F	TTTTTCCCTCGATCTCCGCG	*Vap1*	VAP1_F	ACCCAGGGAAAGGTGGAAAA
	Surfin_1.2_R	GGGTTTGGCCGTACTCTACT		VAP1_R	TGATTTTAGTGCTATTTTTACATGCCT
*Hda_*	HDA_F	AATAATGTGGGCGTGCAAGC	*Tra1*	Tra1_F	GGAAAAACAATGGAGCGTGT
	HDA_R	TTCCTTGCATACCACCCGTT		Tra1_R	TCTTTGTCTTCCAACCATTCG
Ep1	EP1_F	ACACAAACTCCTTACATTAGTGTCG	*Gexp5*	Gexp5_F	GTGGTTGTTTGAGAAGTGGTGA
	EP1_R	TTAGTTGCAAAACGTGGGCA		Gexp5_F	ACAGAATCCGTTTGAGATGATGA
Arom	AROM_F	CCAAAACGGGCGTAATGAACA	*Msrp1*	MSRP1_F	TACCAGGTGCCTTATCAAGTG
	AROM_R	GGGTGACTATTCCCATGATCGT		MSRP1_R	CTTGGTTGTGATTCGGTTGATG
Ep2	EP2_F	ATGCGAGAAACATCCAGATGA	*Sbp1*	Sbp1_F	GGCATCTGCAACTACCGAAT
	EP2_R	TTACAATCCGACCTACAAAGACT		Sbp1_R	GCTTGAAAAACCGTCATCGT
Lshd	LSHD_F	GAGAGATACCAGCAGCAGCA	*Kahrp*	Kahrp_F	CATGGTGCAGGCTATTCAG
	LSHD_R	TATGGGTGCGGCGACTATGT		Kahrp_R	TTCACCGTCATTTCCTTCATGC
Surfin 4.2	SURFIN_4.2_F	CTCATAGCGTTGGTGCTGGT	*Ap2-expo*	Ap2-expo_F	TGTTAGCCAAGAACCAGCAG
	SURFIN_4.2_R	ACTTCCTTGCCCACCACTTC		Ap2-expo_R	AAAAAGCTAGCCACGCACAC
Nyn	NYN_F	GGTCATCAAACAGCGTACGT	*Pfs230*	Pfs230_F	GACCATATCAAACAAATACTTCAGGA
	NYN_R	AGCCATTTTTATGAGCAGCTAGC		Pfs230_R	CTCTTGATTGAGGTTCTGGGAT
Rga	GTPase_F	TGGTTACGATCCAACGACGT	*Pfs25*	Pfs25_F	TCTTTTCCTTTTCATTCAACTTAGCA
	GTPase_R	TCCACCACAAAATGTTGGACA		Pfs25_R	CCACTCATCTGAATTAAAAATCCTCTT
Ep3	EP3_F	ACGAAAAGGACAACGAACAATCC	*Pf18s rRNA*	18SrRNA-F	GCTGACTACGTCCCTGCCC
	EP3_R	TTCTTCGGAACAGAACGTCTT		18SrRNA-R	ACAATTCATCATCATATCTTTCAATCGGTA

Supplementary Table 3. Details of primers and probes used in TaqMan RT-qPCR

Gene	Primer name	Oligo Sequence (5' - 3')	Probe Sequence (5' - 3')	Florescent dye (5')	Non quencher dye (3')
Ap2-g	Ap2g_TaqMan_2F	CCGCTAAGATATTAAATCCCTTCGATA	CCCTGTATACCCTTCTTCGAAAGCATGC	FAM	MGBNFQ
	Ap2g_TaqMan_2R	AGTGCTCTCCTTTCGTGTTG			
Surfin 13.1	Surfin 13.1_TaqMan 1F	CATCAAGCTCTTCTGTTCCTCTT	CCCTCATTCTTCAGTTCCTGCATTATCT	VIC	MGBNFQ
	Surfin 13.1_TaqMan 1R	GGGTGGAGTAGCACTATCTGTA			
Surfin 1.2	Surfin 1.2_TaqMan 3F	CTCGATCTCCGCGTATTTACAA	ACGGCCAAACCCTACTCACATAAACA	FAM	MGBNFQ
	Surfin 1.2_TaqMan 3R	GACAAGCTCACTTACCGAATCA			
Sbp1	SBP1_TaqMan 3F	CCGACGAACCAACACAATTAC	TTGGCCGAAGTAGTTTCGGATGCA	VIC	MGBNFQ
	SBP1_TaqMan 3R	ACTAGCTGCTTCTCCAACTAAA			
