# Supplementary material for: The transcriptome of circulating sexually committed Plasmodium falciparum ring stage parasites forecasts malaria transmission potential
Source: Nat Commun. 2020 Dec 2;11:6159. doi: 10.1038/s41467-020-19988-z (PMC7710746; doi:10.1038/s41467-020-19988-z)
Supplement: Supplementary file 2 — Reporting Summary [file 41467_2020_19988_MOESM2_ESM.pdf]

nature research

Corresponding author(s): Surendra K Prajapati

Last updated by author(s): Sep 25, 2020

# Reporting Summary

Nature Research wishes to improve the reproducibility of the work that we publish. This form provides structure for consistency and transparency in reporting. For further information on Nature Research policies, see our Editorial Policies and the Editorial Policy Checklist.

## Statistics

For all statistical analyses, confirm that the following items are present in the figure legend, table legend, main text, or Methods section.

| n/a | Confirmed | |
|---|---|---|
| ☐ | ☒ | The exact sample size ($n$) for each experimental group/condition, given as a discrete number and unit of measurement |
| ☐ | ☒ | A statement on whether measurements were taken from distinct samples or whether the same sample was measured repeatedly |
| ☐ | ☒ | The statistical test(s) used AND whether they are one- or two-sided<br>*Only common tests should be described solely by name; describe more complex techniques in the Methods section.* |
| ☐ | ☒ | A description of all covariates tested |
| ☐ | ☒ | A description of any assumptions or corrections, such as tests of normality and adjustment for multiple comparisons |
| ☐ | ☒ | A full description of the statistical parameters including central tendency (e.g. means) or other basic estimates (e.g. regression coefficient) AND variation (e.g. standard deviation) or associated estimates of uncertainty (e.g. confidence intervals) |
| ☐ | ☒ | For null hypothesis testing, the test statistic (e.g. $F$, $t$, $r$) with confidence intervals, effect sizes, degrees of freedom and $P$ value noted<br>*Give P values as exact values whenever suitable.* |
| ☒ | ☐ | For Bayesian analysis, information on the choice of priors and Markov chain Monte Carlo settings |
| ☐ | ☒ | For hierarchical and complex designs, identification of the appropriate level for tests and full reporting of outcomes |
| ☐ | ☒ | Estimates of effect sizes (e.g. Cohen's $d$, Pearson's $r$), indicating how they were calculated |

*Our web collection on statistics for biologists contains articles on many of the points above.*

## Software and code

Policy information about availability of computer code

| | |
|---|---|
| Data collection | Primer3Plus, QuantStudioTM Design & Analysis Software v1.3.1, Plasmodium genome database (www.plasmodb.org). |
| Data analysis | SPSS version 24, QuantStudioTM Design & Analysis Software v1.3.1, Microsoft Excel 2013, Graphpad Prism 7, MORPHEUS (https://software.broadinstitute.org/morpheus/). |

For manuscripts utilizing custom algorithms or software that are central to the research but not yet described in published literature, software must be made available to editors and reviewers. We strongly encourage code deposition in a community repository (e.g. GitHub). See the Nature Research guidelines for submitting code & software for further information.

## Data

Policy information about availability of data

All manuscripts must include a data availability statement. This statement should provide the following information, where applicable:

- Accession codes, unique identifiers, or web links for publicly available datasets
- A list of figures that have associated raw data
- A description of any restrictions on data availability

All data generated during this study are included in this manuscript and its supplementary information files. Source data are provided as a Source Data file.
RNA microarray data associated with figure 1.
RNA microarray data submitted to Gene Expression Omnibus (GEO) repository (accession number-GSE152536).

# Field-specific reporting

Please select the one below that is the best fit for your research. If you are not sure, read the appropriate sections before making your selection.

☒ Life sciences ☐ Behavioural & social sciences ☐ Ecological, evolutionary & environmental sciences

For a reference copy of the document with all sections, see nature.com/documents/nr-reporting-summary-flat.pdf

# Life sciences study design

All studies must disclose on these points even when the disclosure is negative.

| | |
|---|---|
| Sample size | The required sample size for the field work was estimated using the computer program (FPOWER), available in STATA software |
| Data exclusions | Sample <0.5% parasitemia were excluded from the H- and L- cohorts and the GCR comparison analysis due to the limited sensitivity of the ex vivo microscopy assay. This criteria was pre-defined. |
| Replication | All in vitro experiments were repeated independently 2-4 times. The field samples were tested in multiple independent subjects. |
| Randomization | All samples were included in the assays, except for the microarray analysis and validation of microarray data (fig. 1 & 3). These sample sets were not randomized so that a range of D8 GCRs would be included and the D0 parasitemias of the H and L-GCR populations could be matched as well as possible. |
| Blinding | High and low GCR groups samples were blinded for microarray analysis which was normalized during analysis. The rest of the assays were not blinded because the results were objective, not subjective, measurements and therefore less subject to investigator bias. |

# Reporting for specific materials, systems and methods

We require information from authors about some types of materials, experimental systems and methods used in many studies. Here, indicate whether each material, system or method listed is relevant to your study. If you are not sure if a list item applies to your research, read the appropriate section before selecting a response.

## Materials & experimental systems

| n/a | Involved in the study | |
|---|---|---|
| ☒ | ☐ | Antibodies |
| ☐ | ☒ | Eukaryotic cell lines |
| ☒ | ☐ | Palaeontology and archaeology |
| ☒ | ☐ | Animals and other organisms |
| ☐ | ☒ | Human research participants |
| ☐ | ☒ | Clinical data |
| ☒ | ☐ | Dual use research of concern |

## Methods

| n/a | Involved in the study | |
|---|---|---|
| ☒ | ☐ | ChIP-seq |
| ☒ | ☐ | Flow cytometry |
| ☒ | ☐ | MRI-based neuroimaging |

## Eukaryotic cell lines

Policy information about cell lines

| | |
|---|---|
| Cell line source(s) | The following reagent was obtained through BEI Resources, NIAID, NIH: Plasmodium falciparum, Strain NF54 (Patient Line E), MRA1000, contributed by Megan G. Dowler. Pfgdv1.gfp.dd was produced in our laboratory and gametocyte parasite lines (683, 565, 592, 607) were also generated in our laboratory. PfE5.ap2-g.dd line was provided by Prof. Manuel Llinas (Penn State University, USA) |
| Authentication | Genomic DNA from the parasite lines were analyzed for the presence or absence of the appropriate chromosomal insertions/ modifications/alleles. These parameters were reassessed periodically throughout the study. |
| Mycoplasma contamination | None of these line were tested for mycoplasma contamination. |
| Commonly misidentified lines (See ICLAC register) | None |

# Human research participants

Policy information about studies involving human research participants

| | |
|---|---|
| Population characteristics | There were three distinct populations of human subjects-<br>1) Subjects (ages 0.6-13 years of age) with uncomplicated malaria (parasitemia ranging from 1000-250,000 per µl of blood) attending EWIM clinic in Cape Coast ,Ghana A blood sample was taken prior to treatment with a standard curative dose of artemether-lumefantrine (20/120 mg/kg) or artesunate-amodiaquine (4/10 mg/kg) (Usui et al 2019, Nat Comm). A fraction of the blood sample was stored for RNA isolation, Hb levels and WBC counts, and two separate aliquot were cultured for 8 in the presence of N-acetyl-glucosamine to block asexual replication. Samples from the ex vivo culture as well as the D0 blood sample were used in this study.<br>2) Adult volunteers participating in a control infection study (VIS) that was published earlier (Collins et al 2018 J Clin Invest). RNA available from blood samples collected from 4 volunteers on the day of peak parasitemia prior to drug treatment and on day 18 was used for additional gene expression analysis.<br>3) Healthy School children (ages 5-13) participating in a cross sectional study evaluating asymptomatic P. falciparum parasite carriage in Ghana had blood sample taken, |
| Recruitment | Ex vivo Study Patients (age- <13 year) visiting the clinic were screened for malaria by microscopy. Following a positive diagnosis of uncomplicated malaria and the child's parent or guardian was asked if they would be willing to have their child participate in this study. If they agreed the study was explained in detail and they were asked to give informed consent.<br>Asymptomatic subjects were recruited from children attending school in Siwim. Prior to the collection day, their parents or guardians were asked if they would be willing to have their child participate in this study. If they agreed the study was explained in detail and they were asked to give informed consent. The VIS subjects had been recruited for a previous clinical trial.<br>-All the available samples were included in our analysis, however none of the subjects had severe malaria and came from a small region of Ghana or Australia so the results may not reflect other populations. All the samples were also from peripheral venous blood and may not be relevant to parasites in the skin or tissues. |
| Ethics oversight | Uniformed Services University of the Health Sciences (USA), Noguchi Memorial Institute for Medical Research and University of Ghana, Ghana Health Services (Ghana), National Institute of Allergy and Infectious Diseases (USA) and QIMR Berghofer Medical Research Institute Human Research Ethics Committee (Australia). |

Note that full information on the approval of the study protocol must also be provided in the manuscript.

# Clinical data

Policy information about clinical studies

All manuscripts should comply with the ICMJEguidelines for publication of clinical research and a completedCONSORT checklist must be included with all submissions.

| | |
|---|---|
| Clinical trial registration | ClinicalTrials.gov NCT02431637 and NCT02431650 |
| Study protocol | https://doi.org/10.1172/JCI98012DS1 |
| Data collection | The initial clinical trial collected blood samples for 38 days after inoculation with blood stage P. falciparum parasites. |
| Outcomes | This work was not part of the original clinical trial, which was published in 2018, Collins et al J Clin Invest. We obtained ethical approval to reuse the available RNA samples for this discovery study. |
